# Spirit of the British and American Periodicals

**Published:** 1841-04-01

**Authors:** 


### Spirit of the British and American Periodicals &c.

---

### Gangrena Senilis, or Pott's Gangrene.

In the first number of a new contemporary (Edinburgh Monthly Journal of Medical Science,) Mr. Syme has published some surgical cases, of which we shall here notice the subject at the head of this paper. The gangrena senilis has been too often considered one of the opprobria of physic, as well as of surgery. It is generally fatal, under the ordinary treatment of stimulants, nourishing diet, and opium. The age of the patients, the circumstance of the vessels having been often found ossified, the great debility of the subjects, and other circumstances, have naturally led practitioners to adopt the stimulating treatment; but it has not been successful, and Mr. Syme has, we think with much judgment, tried a very different practice.

"Although the local soothing plan advocated by Mr. Pott alleviates the patient's sufferings and delays the progress of the disease, it never, in any instance that has fallen within my observation, proved sufficient to arrest completely the mordid action. In order to attain this more important object, it is necessary to lower the tendency to excitement throughout the system, by enforcing a strictly vegetable diet, abstinence from every sort of stimulant, and the maintenance of perfect quiet in the horizontal posture."

Mr. Syme is aware that this plan is likely to meet with opposition, both from patient and practitioner, as it runs counter to the prejudices of the one, and the theory of the other. But, in cases so avowedly hopeless, it is our duty to abandon established routine, when it is found to be so generally ineffective. We shall abridge the first case which Mr. Syme has adduced.

Helen Byres, aged 57, but appearing very much older, was admitted into the hospital, 28th January, 1840, complaining of severe pain in her left foot, especially the great and little toes. The instep was red and somewhat swelled—little toe black, and great toe of purplish dark colour. She attributed her complaint to exposure to cold, and the pressure of a shoe. She had been a week in hospital before Mr. Syme's notice was attracted to the case. During that time she had wine, nourishing food, &c. but the malady progressed.

"Having ascertained the nature of the complaint, I did not hesitate to order a strictly farinaceous diet, water for drink, and a simple poultice for the foot. The symptoms then gradually abated, and the patient, instead of sinking under the united effect of disease and weakness, as she had previously threatened to do, acquired additional strength, and greatly improved in her appearance. In the beginning of March the little toe separated at its metatarsal joint, and about three months afterwards the great toe did the same. The sores healed kindly, and presented on each side of the foot a no less seemly cicatrix than if a skilful amputation had been performed. The starving plan was then abandoned; and the poor old woman, after subsisting on bread and water for upwards of four months, was allowed the usual diet of the hospital."

While we assent to Mr. Syme's experiment of treating the gangrena senilis, or gangrene of Pott, we must say that the case above narrated does not appear to be a severe example of the disease, but rather one of chilblain, or frost-bite, in an aged, weakly, and bad constitution. The following passage is worth extraction, as most surgeons must have met with examples of the same kind.

"In illustration of the treatment which it is my present object to recommend, may be mentioned a case by no means rare in private practice. The patient is usually a man in easy circumstances, somewhat addicted to the pleasures of the table, and beyond sixty years of age. Without any warning he observes a small pimple on his leg. It opens, and leaves a small sore, which, instead of healing, becomes covered with a slough, generally of a black colour, but sometimes white. The surrounding skin now inflames to a small extent; pain gradually increases, and is felt most severely at night, so that sleep is disturbed or prevented. The system then becomes seriously deranged, and the local affection still increasing, there is no limit to the morbid process except death itself.

The tendency to mortification in this form of disease, just as in that so well described by Mr. Pott, leads practitioners to the employment of invigorating measures. And I have uniformly observed, that whether the patient was stimulated by an additional allowance of food and wine, or was permitted merely to continue his ordinary diet, the sloughing action prevailed in opposition to every sort of soothing application that could be tried locally. But when the starving plan was adopted, and the patient restricted to vegetable articles of support, the redness has quickly disappeared, the pain has gradually decreased, and the sloughs, ceasing to extend, have been detached from a subjacent healing surface of granulation, which before long formed a sound cicatrix. The only means employed on such occasions, in addition to the vegetable regimen, have been linseed poultices, and the muriate of morphia given freely, either solid or in solution, so long as the nocturnal pains continued. It may be added, that no inconvenience has ever been sustained, to my knowledge, either from adopting the spare system, or resuming the ordinary one, even when the age of the patient was beyond eighty years."

We return Mr. Syme our thanks for this useful and practical hint. Other cases will be noticed in our Clinical Review.

---

### LARYNGISMUS STRIDULUS.

Dr. Henderson, of Edinburgh, has written a paper in the first number of our new contemporary, (Ed. Monthly Journal,) on the above subject, chiefly as a critique on the late Dr. Ley's work on the same subject. It is unnecessary to notice Dr. H.'s criticisms, as very few practitioners in this country look on Dr. Ley's theory as more than an ingenious physiological romance. The following fact is interesting.

A female, aged 52, was admitted into the Infirmary, 27th August, 1839, who had become affected with a cough and difficulty of breathing, five weeks previously, after working in a damp place. The dyspnœa had increased three weeks before admission, with pain in the front of the neck. At the time of admission the respiration was stridulous, with prolonged inspirations—pain on pressure in the throat—barking cough—scanty expectoration—pulse 108, and small. Twenty-four leeches to the neck. 28th. The breathing is calmer, but of the same character—painful deglutition—pulse 144. Venesection to sixteen ounces. Calomel and opium. 29th. Blood buffed—dyspnœa increased. Tracheotomy, was performed by Mr. Syme, with immediate and great relief. She lingered till the 5th September, when she expired.

*Dissection.*—A considerable quantity of thin puriform matter was found infiltrated into the cellular tissue behind the pharynx and œsophagus, and along the inner aspect of the carotid arteries, enveloping the recurrent nerves of both sides, so that the whole cellular tissue in front of the vertebræ, behind the œsophagus, and between the sheaths of the carotids, was occupied with pus. The larynx was sound; so was the trachea, and neither of them was compressed by the effused matter. There was some purulent effusion in the lungs.

"That the difficulty of inspiring in this case resulted from an obstruction in the glottis, is abundantly evident, both from the descent of the larynx at every attempt to expand the lungs, and from the relief which followed from the opening of the windpipe. That a rigid contraction, or spasm, of the laryngeal muscles was the immediate cause of this obstruction, is proved by the circumstance that expiration was not entirely unobstructed, as appears from the noise emitted from the larynx during the passage of the air outwards, an occurrence which is the reverse of that which happens when expiration is free, as in the case of paralysis of the muscles of the larynx; and it is also further proved by the nature of the cough, and by the acuteness of the voice."

---

### Operations for Stammering.

The surgical cure of squinting has been followed by the still more extraordinary operation for stammering. We shall, on the present occasion, merely place before our readers the two following articles, to which a third might have been added from the pen of Mr. Bennett Lucas, who performed the operation in question, and with apparent success, at the Free Hospital, Greville Street.

### Stammering.

*To the Editor of the Medical Gazette.*

Sir,—I beg to submit to your notice a brief statement of my recent proceedings relative to the cure of stammering, premising that I am at the present time actively engaged in collecting materials for a more lengthened explanation of my views upon the subject.

In the practice of my department of the profession, it has been usual with me to explore the condition of the mouth and pharynx in every case of deafness committed to my care. I have thus discovered that a large number of patients suffering from deafness are affected with enlargement of the tonsils and uvula, and an irritable condition of their investing membrane and the pharynx generally. It has been my constant practice, when I have considered these states at all contributing to the imperfection in hearing, to remove either the tonsils or the uvula, or portions of both, according to the nature of the case, with the most marked and immediate benefit, as far as the hearing may have been concerned. In December last it occurred to me to operate in this manner on two patients. They were, at the time of treatment, so deaf, that I did not then address my questions particularly to them, but to their parents, so that I was unaware of any impediment to speech in these instances. Some time after, as the cure of deafness advanced, I learned from the parents that both children had been stammerers from infancy, and, as much to my surprise as gratification, that the cure of stammering had ensued immediately on the excision of the tonsils. At the time at which I write, the subjects of both these cases remain free from any impediment, though their stammering previously to the operation is represented to me as having been very decided. I had before this remarked that persons with enlarged tonsils were affected with thick and imperfect speech, for which I had often, during the last year, practised excision with the happiest effect, in restoring the voice to its original clearness. Since the cases above-mentioned, I have operated on upwards of forty persons, all of whom have immediately felt themselves relieved of their impediment. Many have seemed wild with joy, or have shed tears of pleasure, at the instantaneous restoration they have enjoyed. After the operation, the difficulty of speech which remains is referred by the patient to the lips; they express themselves entirely free from the original difficulty. Something must be allowed for the long misuse of the organ of voice, and the existence of habit, in rendering the voice less perfect than in the natural state. In fact, after their relief, patients have yet to learn the proper use of the vocal apparatus.

I have performed the operation by means of a scalpel, tenaculum, and scissors, without any serious hæmorrhage, and with a small amount of pain, which has appeared somewhat greater in the case of the uvula than the tonsils.

In reflecting upon the subject, the explanation I have at present to offer is, that to produce stammering, the dorsum linguæ, the palatine arches, velum palati, and uvula, approximate together so completely, and perhaps irregularly, as to leave no room for the expulsion of air from the larynx. In a person who stammers, no air issues from the mouth during the abortive effort to speak; but it does so as soon as the patient is relieved from this state, so as to produce sound. The most violent contractions of the abdominal muscles can be seen attempting to force up the diaphragm and expel the air; sometimes all the respiratory muscles, and even those of the body generally, are thrown into violent spasmodic action, as the individual grasps some near object to assist the expulsive effort. In some cases, when there is nothing abnormal about the tonsils or uvula, I find a great congenital narrowing of the entrance from the mouth to the pharynx.

I submit that the operation, which I believe I am the first ever to have proposed or performed specially for the cure of stammering, relieves this malady by making, as I excise the tonsils or uvula, an opening in the valvular obstruction I have described as being formed by the joint agency of the tongue, palatine arches, and soft palate. You will perceive that the principle of my operation is quite different from that ascribed to M. Dieffenbach, and since practised, it is said, with success in France, by MM. Amussat and Phillips, and in this country by Mr. Bennett Lucas. This operation appears to me to rest upon the principle that the tongue is chiefly concerned in the production of the voice; whereas, I would inquire, does not this organ rather serve to modulate the voice after it has issued from the pharynx, &c. while in stammerers it is the fresh production of sound which creates the difficulty, rather than its subsequent modulation?—I am, sir,

Your obedient servant,

JAMES YEARSLEY.

29, Sackville Street,

March 9, 1841.

### AN ACCOUNT OF DIEFFENBACH'S NEW OPERATION FOR THE CURE OF STUTTERING.—(With Case.)

*To the Editor of the Medical Gazette.*

SIR,—We have lately read, in the daily papers, several paragraphs from correspondents, concerning a new operation for the cure of stammering. The French papers have likewise furnished us with accounts of it. But it seems that the English journalists merely copied the French, who obtained their information from the German daily papers, which, indeed, stated the effect of the operation correctly, but described the manner of performing it very imperfectly, and without entering into the particulars at all, as I found on reading them three or four weeks since. The same remark has been made by M. Velpeau, in a discussion which took place at the Academy of Medicine on the 16th of February. It is, therefore, not surprising that the English and French journals should describe incorrectly the nature of the operation, and the manner of performing it, as practised and originally proposed by its discoverer. It is to that bold and distinguished surgeon, Dieffenbach, who lately enriched surgery with that beautiful operation for strabismus (which I first performed in Great Britain, as is decisively evident from my publications, and the testimony of several periodicals,) that we are again indebted for the discovery of a cure for stuttering. On the 22d of last month I received from my friend, Professor Dieffenbach his memoir on this subject, as addressed to the Institute of France, and dated the 31st of January, 1841. From the information obtained from this source I was enabled to perform this new operation according to the rules laid down by him, an account of which operation, as well as the leading points of the professor's letter to the Institute, are laid before the profession in the following paper; and if you can find room for it in the next number of your valuable journal, you will greatly oblige, sir,

Your obedient servant,

19, Golden Square, AUG. FRANZ, M.D.
March 9, 1841.

Professor Dieffenbach says, in the above-mentioned memoir, that " the infirmity of stuttering had long engaged his attention, but its probable cure suddenly occurred to him while a patient who had come to him to be operated on for strabismus addressed him with stammering accents. He was led to the idea of removing this impediment by an operation, from observing that the nervous twitchings of the eyelids, and spasms of the muscles of the face, which frequently accompany squinting, immediately disappear after the division of the muscles of the eye. He had frequently seen that by dividing the frænum linguæ, in cases where it was too much tied down, as also by other operations on the tongue or on the soft parts situated at the back of the mouth, some improvement was obtained in those cases where there was a slight hesitation in speech, but never in actual stuttering. He therefore reasoned that in this infirmity the disturbance in the mechanism of speech must originate from a dynamic cause; viz. from a spasmodic state commencing in the trachea, but more especially in the rima glottidis, from whence it extends to the tongue and the muscles of the face and neck: consequently he came to the conclusion that an interruption of innervation in one of the parts affected might be followed by an alteration of the nervous influence, and therefore by a suspension of the disturbance in the mechanism of speech. He thought that this alteration would be most certainly obtained in the nervous action of the vocal organs, by dividing the root of the tongue right across, and through its whole thickness; for doing which he has proposed three different methods:—

1st. The horizontal transverse section of the root of the tongue;

2d. The subcutaneous transverse section of the root of the tongue, with preservation of the mucous membrane;

3d. The horizontal transverse section of the root of the tongue, with excision of a portion in the form of a wedge.

The Professor has operated in two cases according to the two first and more easy methods, but finding that although the patient was able to pronounce a few words without stammering immediately after the operation, yet on its recovery the impediment was not entirely removed, he does not speak in such high terms of these as of the third method. According to the latter he has performed fourteen operations: the earlier cases, he states, are perfectly cured not only of the stammering but also of the spasmodic contractions of the muscles of the face, neck, and thorax, and the latter cases promise an equally favourable result. He therefore pronounces this method of performing the operation, notwithstanding its severity, as far superior to the two former, ' since by cutting out a transverse portion of the tongue the alteration of the nervous action on the vocal organs must be more decided; the tongue likewise becomes shortened, and its tip turned upwards, which latter circumstances has always been considered as contributing materially to the removal of stammering.'

The Professor further hints, that ' in selecting a case for this operation, the greatest circumspection is required; that in the execution of it, great dexterity and quickness are necessary, on account of the situation of the tongue, and the enormous hæmorrhage which of course takes place: and that in the after-treatment peculiar care is to be observed. If a strict attention is not paid to these circumstances, this operation, which inflicts so great a lesion on an important organ, may either be followed by destruction of the tongue, or even by loss of life.' The first operation for the cure of stuttering was performed by the Professor on the 7th of January, 1841, on a young gentleman 13 years of age, and was followed by a perfect cure. In this case, he excised a piece, of the shape of a wedge, from the root of the tongue, being three-quarters of an inch at its superior part, and extending through its entire thickness. I shall not enter here into the details of the operation, as they will be seen from the following case, operated upon by myself, according to Dieffenbach's third method.

*Case.*—George Read, 17 years of age, of a strumous habit, but otherwise strong and healthy, except an herpetic eruption upon the lips, with which he has been afflicted some time. His parents, brothers, and sisters, were not afflicted with any impediment in their speech, neither was the patient so up to his father's death, which took place eight years ago, and made such an impression on his mind, that immediately after its occurrence he began to stutter. This infirmity, according to his mother's account, has gradually increased up to the present time; so that now he is scarcely able to speak, even to his own family, without great hesitation, though composed and free from excitement in mind and body. He, however, succeeds best in expressing himself by singing in a high pitch what he has to say. When he is in the slightest degree agitated, he can with difficulty stammer out one or two words only, and then his speech becomes convulsively interrupted, and, ultimately, entirely stopped; but when in the least excited, if he attempts to address a stranger, speech entirely fails him. When he first called on me, with his mother, his countenance exhibited the utmost degree of melancholy, the result of this trying calamity. His palate is highly arched, and rather narrow; his tongue could be well raised, and freely moved in the mouth. He aspirated all the vowels, and pronounced them thus —h-h-h-a, whe, whi-hi, who-h-o-o, whu-h-u-h-u; consonants whose pronunciation commences with a vowel, he pronounced thus—hhell, hheff, hhess, &c.; the other consonants he pronouced with more or less difficulty; but d, t, b, and p, he could not pronounce at all. When he attempted to speak, he thrust his head forwards; moving it from side to side; his lips were drawn together, and protruded; and the muscles of the mouth, at the same time acting convulsively, drew them in every direction. The alæ nasi moved as in a paroxysm of dyspnœa. The eyes started forward, and began to water. The muscles of the face were in convulsive motion, as also those of the neck, although in a less degree. The larynx was drawn upwards. His mother informed me that, when in this state, he experienced a sensation of rigidity of the tongue, and as if it were too large for his mouth; as also of constriction of the muscles of the neck, but more especially of those of the larynx. I wished to make him speak a few words; but while attempting to do so, his eyes protruded, tears ran down his cheeks, and the convulsive movements of the muscles of his face and neck, but especially of those of the mouth, became so violent, that he was obliged to desist. During the whole time he was with me, the only word he could in any way pronounce was the word no, which he did as if written thus—h-h-n-nho-o. As he was utterly unable to answer any question, I was obliged to have recourse to his mother, as interpreter, who knew from the motions of his lips what he wished to say.

On my explaining to them that a new operation for the cure of stuttering had been tried, and had succeeded in many instances, they readily consented to have it performed, as his stuttering to this dreadful degree, almost equivalent to dumbness, rendered him exceedingly wretched in mind, incapacitated him for business, and cut him off from all intercourse with any but his relatives.

On the 1st of March I performed the operation at the mother's house, in the presence and with the kind assistance of Dr. Ure, Messrs. T. Fowke and W. Hering, who before the operation satisfied themselves of the patient's utter inability to pronounce the simplest word. The patient was seated in a high chair opposite the window; his head being held in a perpendicular position by one assistant, who, at the same time, drew the angle of the mouth backwards by means of retractors. His tongue being thrust forwards was seized by a pair of strong forceps, (furnished with teeth to prevent them from slipping,) which I gave to another assistant to hold; by these means the tongue was steadied and compressed transversely. Having seized the tongue, posteriorly to the forceps, with the thumb and forefinger of my left hand, I compressed it transversely, and at the same time elevated it; then passing a long, curved, pointed bistoury, from the left side beneath the tongue, and at the posterior half, until I felt the point at the right side with my fore-finger, I cut directly upwards, dividing the tongue right through. I now grasped the tongue in front of the wound with a pair of long forceps, armed at the point with teeth, pressed it firmly together, and with a small straight scalpel made a section from above downwards, commencing on the dorsum linguæ about half an inch anteriorly to the division already made, and meeting it inferiorly; by these two sections a piece of the shape of a wedge was cut out of the tongue at its posterior half. The wound was now united by six ligatures of thick silk, which were passed in such a manner as to encircle in depth and breadth a considerable portion beyond the margin of the wound, and were forcibly drawn together in order to restrain the hæmorrhage; this was so far successful that only a slight oozing continued for a short time. That the loss of blood during the operation was very considerable is not to be wondered at, when we consider the size and number of the blood-vessels divided. The patient bore this severe operation well, but became rather faint towards the termination, and afterwards vomited large quantities of blood which he had swallowed. As soon as he had washed his mouth with a little water, I was exceedingly pleased to hear him pronounce words which, previously to the operation, he was utterly unable to articulate; such as time, powder, &c., without the slightest hesitation or stammering, and without any twitchings of the lips, or even convulsive movements of the muscles of the face or neck, and immediately afterwards I was surprised by his saying with facility and distinctness, "there is some blood running down my shirt." He was now put to bed, and desired to be kept quiet, and directed not to be allowed to speak, and to have his mouth kept cool by means of cold water. On my calling in two or three hours time I found the case proceeding favourably; no re-action had as yet taken place.

March 2nd.—The patient had slept well during the night. Some febrile action towards night; pulse 120, but skin moist. On examining the mouth I found the tongue somewhat swollen and discoloured; he complained of no pain, but only a feeling of heat in his mouth: there was a great secretion of saliva and mucus, and some difficulty in swallowing. He took, during the day, a good deal of beef-tea, and towards evening a dose of castor-oil. Was ordered to continue the application of cold water to the mouth.

3d.—He passed a very good night; was almost free from fever. The pulse had fallen to 95. Tongue rather more swollen; not painful, but thickly covered with a brown substance. To take a saline mixture every three or four hours.

4th.—Still going on well. Pulse 80. I now removed two ligatures, and found that union had taken place.

5th.—Swelling of the tongue greatly abated; but the copious secretion of saliva and mucus still continuing as before. He was now able to swallow liquids with but little difficulty, and was so far recovered as to sit up for a short time. He spoke a sentence or two without the slightest hesitation or stammering, and without any convulsive motions of his lips or face. The remaining four sutures were removed, after which, by cleaning the mouth with some water, a great deal of the brown substance before mentioned came off, leaving the mucous membrane perfectly clean and healthy; in the neighbourhood of the wound, however, it remained adhering to the tongue.

7th.—The tongue still continues clean; can be moved freely, but not without some degree of pain: speaks without stammering.

9th.—Has for the first time taken a walk in the open air. The movements of the tongue less painful. The mother gives a favourable account of the progress of his speech.

Perhaps it may be as well to state that the muscles divided in this operation are the lingualis, the genio-hyo-glossi, the hyo-glossi, and the stylo-glossi.

I think, previously to undertaking this operation, we ought to consider well whether the nervous system of the patient is sufficiently strong to stand so severe a shock—whether he will, from age or constitution, be able to bear so considerable a hæmorrhage as that which is here inevitable; and it would be advisable likewise to ascertain that the heart and large blood-vessels are in a healthy state. The further remarks I had purposed to make on this important operation will be appended to another paper, in which I shall, on a future occasion, communicate to the profession the final result of the above case, as also the first and second methods of performing Dieffenbach's operation for stuttering, which I have not yet described. But I cannot conclude this account without returning my thanks to my friends, who took great interest in this case, and kindly assisted me through it."*

### Protracted Gestation.

In a communication of some "cases in legal medicine" to the American Journal of the Medical Sciences, by Dr. Beck, we find the following.

*Case.*—Mr. and Mrs. G—— are Polish exiles of excellent education and morals, and highly esteemed among their acquaintances.

Some time in February, 1840, Dr. Manley was requested to take charge of Mrs. G., as she expected to be confined about the 10th or 15th of April ensuing. She had previously been his patient, having treated her for hæmoptysis. On the 7th of April, however, he was sent for to prescribe for another and violent attack of the same complaint. She was bled twice largely, kept on a low diet, absence from all stimuli, even light, &c., for about three weeks, in the hope that her daily expected confinement would be her chief security against the ordinary consequence of her disease, viz. consumption. The treatment was successful, the cough and hæmorrhage ceased, but the parturient effort was deferred until the 29th May.

This circumstance created much surprise and anxiety in the minds both of her friends and her physician. The latter, upon close inquiry, satisfied himself of

* Med. Gazette, March 12, 1841.

the following facts. 1. Her husband had left her on business on the 13th of July, 1839. 2. He did not return until the last of November. 3. During the first three months of pregnancy, she was twice unwell or menstruated, but as she was 39 years old, and had borne six children, and the amount was trifling, she, in place of deeming herself pregnant, thought it furnished evidence of the approaching cessation of the function, and particularly as the ordinary accompaniments of previous pregnancies, viz. sick stomach, toothache, &c., were altogether wanting; but 4. On the 30th of November, she felt quickening, which, allowing four and a half months for the mean time of the appearance of that sign, made her reckoning for the 15th of April correct. 5. On the nights of the 12th and 13th of December, she was attacked with hæmoptysis, for which Dr. Manley treated her by bloodletting and the negation of stimulants and nutrition, and was successful. The occasional hæmorrhage continued however for nearly three weeks. 6. On the 7th of April, as before observed, she was again seized and treated successfully. This was a violent attack, and the treatment was carried to a greater extent than before.

"About the 16th of April, having occasion to leave home, I put the patient under the care of my friend Dr. Duvall, in whom I could place the utmost confidence, believing that she would be confined every hour, as she complained of pains, which I presumed were premonitory, though not parturient. When I returned after an absence of three days, I found things as I had left them. She complained of much uneasiness *in perinæo*, and told me that Dr. D. had ordered an enema, which could not be administered from inability to insert the pipe of the instrument; said that she was sitting upon the child—that if she got up and again sat down, she felt unpleasant, as if something was pressed upwards. About the first of May, so much anxiety was manifested by her friends, that without my knowledge, Dr. S. was requested to visit her, and after examining into her condition, was sufficiently satisfied to pronounce that she was not pregnant *with a child*, although she might have hydatids or a mole *in utero*. As soon as I was informed of this opinion, I lost no time in satisfying myself concerning her condition. I made a thorough examination by auscultation and the sudden application of the cold hand to the abdomen. The result was such as could not deceive, and I pronounced her pregnant and near delivery, which might take place within two hours, as the head of the child rested, I may almost say, on the perinæum, and the os tincæ was dilated larger than a dollar. The head was so wedged, that every attempt to pass by the side of it produced excessive pain, although the patient complained of nothing but pain in the pelvic region. This particular examination was made about the 6th or 8th of May. She continued in this state, enduring more or less pain every day, till the 29th, when she was happily delivered of a son weighing 9¾ pounds."

There was nothing peculiar in her parturition, except that it was a forehead presentation, and the cord was so convoluted, and entangled the child so much, as to leave but seven or eight inches of its length, being twice round the neck and once round the abdomen. When the waters burst, they came away with a great deal of meconium—calculated to lead to the impression, that there were twins in utero, and that the membranes of each had ruptured. The posterior fontanelle was obliterated, and the anterior was very small, not exceeding the size of a five cent piece. The child was remarkably strong, and the papillæ of what is denominated red gum, were in many places marked by yellow points, as is usual, when children are a week or more old. The labour was short, but excessively painful, by reason of the presentation, and the placenta was detached at the time of birth.

After inquiring—can diseases, or the treatment necessary for their cure, have an influence in protracting the term of gestation? Dr. Manley argues in the affirmative, and concludes that it appears that Mrs. G. carried her child 10 months and 13 days; but this admits of easy explanation, if we allow the treatment which was rendered necessary during her two attacks of hæmoptysis to have had any agency in suspending the process of fœtal growth and uterine evolution; for after all is said, it is not certain that any other cause than the maturity of the fœtus determines the natural period of gestation.

He asks a second question,—may not protracted gestation be often confounded with protracted parturition, and be the cause of many of those anomalies which the subject of gestation presents? This too he answers in the affirmative, and continues—The appearance of the child at birth; its size and weight: the obliteration of the posterior fontanelle, and the very small aperture of the anterior one; the appearance at birth of that peculiar eruption denominated the red gum, (*strophulus intertinctus,*) and the matured character of it, for the vesicles were filled with an opaque yellow serum, all render it probable that the infant was detained in utero beyond the usual time. He adds—

"I instituted no special examination, till I was informed that a surgeon had visited my patient and pronounced that she was not pregnant with a child.—It was early in May, that I made this examination and I found the *os uteri* much dilated and the head of the child resting *in perineo:* no labour pains had been felt, nor did pains of any kind which could be mistaken for labour occur till the morning of the 29th of May, about three weeks afterwards. The labour, however, was a painful one and continued five or six hours, which I attributed to the position in which the child presented. If the presentation had been of the vertex, I doubt not that she would have been confined in a short time and with little suffering: and I cannot doubt, that but for the same cause, she would have been delivered three weeks before. In this event, neither her labour, nor her gestation would have had any extraordinary features, but would have passed, either entirely unnoticed, or would have attracted very little attention."

The case is an interesting one, and we leave it to our obstetric friends. It bears on a very important point of jurisprudence.

---

REMARKS ON THE SOURCES OF HÆMORRHAGE AFTER LITHOTOMY. By JAMES SPENCE, Surgeon; Assistant-Demonstrator of Anatomy in the University of Edinburgh, &c.*

Mr. Spence's paper is likely to be of service. It exhibits the anatomical distribution and varieties of the vessels that *may* bleed in lithotomy, and sets the modes in which the hæmorrhage may happen in the very clearest light. His dissections have been confined to the parts concerned in the lateral operation, and, during the last three years, he has made seventy-three for this purpose. He begins with the—

*Internal Pudic Artery.*

The pudic, in its normal course, can scarcely be wounded posteriorly. But as it passes forwards into the anterior part of the perineum, it gradually leaves the protection of the ramus of the ischium, and where it gives off the artery of the bulb lies between the layers of the triangular ligament. This is the point where the pudic is in danger of being wounded by the gorget—the beaked knife, or lithotome caché, for in using these instruments, the surgeon, after inserting the beak into the groove of the staff, depresses the handle of the staff, and, by a simultaneous movement of his right hand, thrusts the gorget or lithotome along the groove. The consequence is, that the point of the staff being thus elevated, the cutting instrument is guided along it into the anterior and narrow portion

---

* Edinb. Monthly Journ. of Med. Science, No. III.

of the perineum, the prostate is divided transversely, and the pudic artery is in great danger of being wounded on withdrawal of the instrument.

The internal pudic was wounded by the celebrated Desault, who succeeded in tying it. Deschamps, however, seems to doubt that the wounded vessel was the pudic trunk. It was wounded in one case by the late Sir Charles Blicke, where Mr. Abernethy subsequently secured it by ligature. Sir Benjamin Brodie mentions having tied the pudic in a case operated on by the late Sir E. Home. Dr. Physick of New York wounded it in his first operation, on which occasion he used the cutting gorget; in this case the artery was also tied successfully. Mr. Lowe Wheeler mentions three cases in which he had seen this accident occur— one in Paris and two in London; in the first the lithotome caché was used to divide the prostate; in the other two, Blizard's beaked knife. Mr. Crosse of Norwich, whose experience and dexterity in this operation are well known, also relates a fatal case of this nature in his own practice.

"With such cases," says Mr. Spence, "before us, we should be exceedingly cautious in pronouncing any opinion as to the accident being the fault of the operator. It is, however, worthy of remark, that in all the cases mentioned, with the exception of that by Mr. Crosse, who does not give the particulars of the operation, the instruments used to divide the prostate were either the cutting gorget, Blizard's beaked knife, or the lithotome caché; the method of using which, and the danger arising from it, I have already explained. And after mature consideration of the surgical relations of the vessel, and of the cases in which it has actually been wounded, I cannot help thinking that if the operation be performed with the knife, the staff held steady by an assistant from first to last, and the prostate divided obliquely downwards and outwards, wound of the pudic artery will be a rare occurrence indeed."

*Irregularities of the Internal Pudic.*—"I scarcely know of any variety which can be properly termed irregular distribution of the pudic trunk. The nearest approach to such a variety, of which I am aware, is preserved in the Anatomical Museum of the University, and is described by Dr. Monro, in his excellent work on the Pelvis. The irregularity is on the right side. The irregular vessel comes off from the internal iliac direct, passes along the lateral and inferior surface of the bladder, *pierces the ileo-vesical fascia, runs along the lateral lobe of the prostate,* and divides into three branches, one to the dorsum penis, and one to the crus, *whilst the third runs along the membranous part of the urethra to gain the bulb.* Another preparation somewhat similar is contained in the collection of Dr. Allen Thomson, professor of anatomy at Aberdeen, who kindly favoured me with a full description of it, from which the following is an extract:—'It is situated at first between the bladder and rectum, farther down it appears to be on the side of the prostate, crossing it and the membranous portion of the urethra obliquely, before arriving at the subpubic arch, and *quite below the anterior true ligament of the bladder;* when it reaches the subpubic arch, the artery gives downwards a considerable branch, which soon dividing into two, sends one twig into each crus penis. The artery is then continued along the dorsum of the penis as far as the glans.' In a subject which I dissected lately, I found a large vessel arising from the internal iliac in common with the obturator; it then passed along the side of the bladder, and over the upper surface of the prostate; on arriving near the pubic arch it pierced the fascia immediately external to the left anterior true ligament of the bladder, and divided into three branches; one entered the spongy part of the urethra about an inch anterior to the bulb; the other two branches were distributed, one to the dorsum, the other to the crus penis. If such an anomaly as that described in either of the two first mentioned cases, existed on the left side of a person who was to undergo the lateral operation, the artery must inevitably be wounded either in opening the urethra, or on dividing the prostate. In the case which I myself dissected, the vessel would have been in no danger, for it lay completely above the line of incision, and on the upper surface of the prostate.

The variety described by Haller, Burns, Tiedemann, Harrison, and others, which is frequently but erroneously termed irregularity of the pudic trunk, is merely a variety of its terminal branch, the dorsalis penis, and is comparatively common. In my own dissections, I have met with five cases, and I saw another during last autumn in a subject dissected by my friend Dr. Duncan. In all these cases, the course of the irregular vessel was precisely similar: it passed along the lower and lateral part of the bladder, then coursed obliquely across its neck, *above the reflexion of the ileo-vesical fascia,* over the *upper* surface of the prostate gland, and passed out of the pelvis between the anterior true ligaments of the bladder. From a consideration of the relative anatomy of this variety, I think it runs no risk of being wounded, if the operation be performed according to the method now generally recommended, viz., dividing the prostate obliquely downwards and outwards, leaving the neck of the bladder and the reflexion of the ileo-vesical fascia entire, because the vessel runs above the reflexion of that fascia; at least it has done so in every case which I have seen."

The artery in the case of the late Mr. Shaw, a very excellent anatomist and surgeon took the course described, and was cut, and gave rise to fatal hæmorrhage. But Mr. Shaw made an extensive incision, being of opinion that the whole extent of the left lobe of the prostate, and the fascia enveloping it, together with the neck of the bladder, should be freely divided to allow of the easy extraction of the stone.

The *branches* of the pudic are next alluded to.

1. *The Inferior Hæmorrhoidal.*—"In a few cases I have seen the vessel pass almost across the ischio-rectal space without dividing into branches; and as it must always be cut in lithotomy, I should think that in such a case it would bleed profusely, both on account of its own size and its proximity to the pudic trunk. I believe, however, that in most cases the artery could be readily enough secured, unless it be cut so close to its origin as to retract within the fascia, or its coats so diseased as not to hold a ligature, a state in which I have frequently found the hæmorrhoidals in old people; and Mr. Liston mentions a remarkable case of fatal hæmorrhage from this diseased state of the arteries."

2. *Superficial Perineal Artery.*—This is often cut, but, generally, ceases soon to bleed. "M. Roux has remarked, however, that if the surgeon, by lateralizing the knife too much, divide the artery near to its origin, it may retract within the opening of the fascia through which it passed, and by bleeding profusely simulate wound of the pudic trunk. M. Roux, indeed, is of opinion that this is what has happened in all the cases in which the pudic is said to have been wounded from lateralizing too much. Although I do not go so far as M. Roux, I yet believe that his opinion is correct to a certain extent; for when we consider that the artery is stretched during the operation by the scrotum being drawn upwards, we will perceive how apt it will be to retract within the fascia, if divided close to its origin."

3. *Artery of the Bulb.*—Mr. Liston is of opinion that this vessel is in no danger in lithotomy, if the incisions be properly made; but states that it is often cut from want of care; whilst Mr. Key states that although, in experimenting on the subject, he has in general been able to avoid it by cutting very low, he nevertheless believes the vessel must almost always be divided when operating on the living. Sir Charles Bell, after observing that this vessel is often needlessly cut, says, 'Yet it is not easy assuredly to avoid it.' Mr. Crosse thinks the artery of the bulb is frequently cut, and sometimes on both sides. He mentions a case in which the artery on the right side was divided whilst the left was intact. M. Blandin admits that this vessel in many cases cannot be avoided.

"To satisfy myself more fully than I could by ordinary dissection, as to the risk of wounding the artery of the bulb, I performed the lateral operation six times during the session of 1839—40, on subjects previously well injected. In three of these the operation was performed with the curved staff and Mr. Liston's knife; in two with the straight staff; and in one with Scarpa's cutting gorget. In none of these experiments was the vessel actually cut; but in no case was there more than a few lines of substance between the anterior part of the deep incision and the vessel; whilst in one of the cases where the operation was performed with the curved staff, the artery had been just pushed up before the back of the knife, its lower surface being quite bare. From the proximity of the artery to the incision in these and many previous experiments, and from having observed in my dissections of the perineum that the artery of the bulb frequently lies little more than an inch in front of the anus, I am inclined to think that the vessel must be frequently divided in operating on the living. For we cannot well begin our incision lower in the perineum than fourteen lines in front of the anus, otherwise we are in danger of cutting into the groove of the staff through the substance of the prostate, leaving the membranous part of the urethra and the apex of the gland undivided, a circumstance which would cause great difficulty in the extraction of the stone. It may be asked, Why, then, is hæmorrhage so rare. I reply, because in most cases the vessel is divided near the bulb, at a distance from its origin. Again, in some cases the artery is small, and I have several times seen its place supplied by three or four twigs from the pudic. The frequency of wound of the bulb itself, (in which case this vessel must be wounded,) together with an instance related by Sir Astley Cooper, in which he arrested bleeding from the urethra, by cutting down upon and dividing this artery—are farther proofs that it may in some cases be cut without giving rise to profuse hæmorrhage. Indeed, I suspect the greatest risk of bleeding is where the vessel is only wounded, without being fairly cut across."

We quite agree with Mr. Spence. Nothing can be more certain than that cutting too low, to avoid the bulb and its artery, makes the operator liable to open into the staff through the prostate. This has happened to ourselves several times in operating on the dead body. On the other hand, if the incision be commenced fourteen lines anterior to the anus, what *security* can there be against wound of the artery of the bulb?

*Irregularities.*—"In a subject which I dissected in 1837, the artery of the bulb arose from the pudic as usual, but then passed almost directly backwards to near the anus, whence it again curved upwards to gain the bulb. I have also seen two cases similar to that described by Mr. Stanley, in which the vessel came off from the pudic posterior to its usual origin, ran immediately above the inferior margin of the triangular ligament, and then passed upwards to the bulb. It is evident that in such cases, and also where the vessel comes off from the irregular pudic trunk and runs along the membranous part of the urethra, as in the case mentioned by Dr. Monro, of which I have already spoken, this artery must be divided in the lateral operation; and the existence of these anomalies sufficiently disprove Mr. Liston's sweeping assertion, that the artery of the bulb runs no risk, whatever be its course, if the incisions be made low in the perineum."

4. *The Prostatic Artery,* arises sometimes as a distinct branch from the internal iliac, but more generally in common with the vesical, or from the internal pudic, in the first part of its course, before it leaves the pelvis. "In the great majority of cases the vessel passes along the lateral and inferior surface of the bladder towards its neck, then pierces the ileo-vesical fascia and gains the side of the prostate, on which it divides into numerous twigs, which supply that gland and the neighbouring surface of the rectum. In such a distribution of the artery it is not likely to furnish much blood if divided; but in several instances I have seen the prostatic artery gain the perineal surface of the prostate without dividing into minute branches; and in eight of these cases the vessel was fully as large as the artery of the bulb, and would have bled profusely if divided, as it must inevitably have been in the lateral operation of lithotomy. The vessel was noticed by the older surgical authors. Douglas thus speaks of it, when describing the parts cut in Cheselden's operation—" When the prostate gland is divided near the rectum or back part of the pelvis, a large straight arterial branch can seldom escape the knife." Sharpe speaks of applying styptics to " the artery creeping upon the prostate." And more recently, Mr. Carpue speaks of " a branch of the internal pudical artery ramifying on the prostate," and states that he has known " several patients die in consequence of the division of this artery." This statement of Mr. Carpue was severely criticised by a gentleman who denied the existence of any such vessels, and who accused Mr. C. of mistaking the prostatic veins for arteries. Mr. Spence gives a sketch, which is somewhat foggy. We may add that, in two instances, in examining patients by the rectum (one was the subject of calculus,) we have felt a large artery pulsating upon the prostate. It appeared to us that the vessel could hardly escape in the lateral operation of lithotomy.

5. *The Prostatic Venous Plexus.*—It is only of late years that surgical writers have directed their attention to this subject, and even yet they do not seem fully to appreciate the dangers of bleeding from the large veins which are sometimes wounded in lithotomy. For even Sir B. Brodie, in his admirable work on Diseases of the Urinary Organs, in mentioning a fatal case of hæmorrhage from near the prostate says, " and what was remarkable, it was venous," an expression which would lead us to suppose that his attention had then, for the first time, been drawn to this kind of bleeding. Mr. Liston and Mr. Lizars, although they both notice the danger of bleeding from the prostatic plexus of veins, if wounded, seem to think that it should properly not be interfered with. I think this opinion must have reference only to the superior prostatic veins, which, commencing in the dorsal veins of the penis, form a net-work on the upper surface of the ileo-vesical fascia, and on the upper surface of the prostate. But if the plexus be moderately injected from the dorsal veins, and the perineum dissected in the manner recommended when describing the prostatic arteries, it will be found that a plexus of veins, (or in old men rather venous sinuses,) which may be termed the inferior plexus, covers the lower or perineal surface of the prostate. And it is evident that this plexus, from its position, must inevitably be wounded, in making even the most limited incision of the prostate gland. When we consider, moreover, that it communicates freely with the superior prostatic plexus, and inferiorly with the middle hæmorrhoidal veins, and remember the want of valves in these vessels, and their dilated form in old persons, I think it must be allowed, that in them at least, this plexus constitutes a formidable source of hæmorrhage after lithotomy. And of late years it has been noticed as such by Baron Dupuytren, Dr. Monro, MM. Velpeau and Robert, the last of whom has recorded two cases of hæmorrhage from this source."

Thus the artery of the bulb and the enlarged artery of the prostate are the most likely sources of serious hæmorrhage. Mr. Spence has found during his investigations, three cases of irregularity in the artery of the bulb, and eight of the enlarged prostatic artery, so that in eleven cases out of the seventy-three subjects dissected by him, these vessels must inevitably have been wounded. These facts, therefore, are sufficient to prove that hæmorrhage may sometimes occur without any fault on the part of the operator.

A very good paper.

---

### Two Cases of Aneurysm exhibiting the necessity of a Ligature both above and below the Tumour. By W. E. Horner, M.D. Professor of Anatomy in the University of Pennsylvania.*

Case 1.—*Aneurysm from Venæsection—Ligatures above and below the Tumour —Cure.*

Miss B., of Georgia, ætat. 8 years, being exceedingly ill in March, 1837, was bled by a physician in the left arm, at its bend. Nothing unusual at the time was perceived, but in a week afterwards, she felt a small pulsating tumour, the size of a pea: it continued to increase, and she was brought by her parents to Philadelphia, and placed under the charge of Dr. Randolph, who called Dr. Horner into consultation.

At this time, September 27th, 1837, the tumour is about the size of a large filbert: has a strong pulsating motion which may be felt vertically; laterally; and also when the arm is bent, and the tumour pulled up from it. Pressure diminishes its size to one half; it then remains hard and unyielding. Pressure on the brachial artery arrests its pulsation. There is no thrill or purring noise as in varicose aneurysm; the vein which was opened at the point of bleeding, is not visible.

On the 29th of September, an operation was performed by Dr. Randolph, the course of the blood being regulated by a tourniquet on the arm. The skin was slit up for two or three inches in front of the tumour, which exposed the tumour beneath the fascia of the arm and the aponeurosis of the biceps; these being dissected through, the tumour was laid bare by continuing the dissection over its surface, so as to exhibit the brachial artery and vein both above and below it. A ligature common to the two vessels was then carried under them, above the aneurysmal tumour; it, upon trial, was found to control the pulsations of the tumour; it was then fixed, and the aneurysmal tumour cut open. Upon slacking the tourniquet, blood issued from the tumour freely; a ligature was then fixed upon the artery and vein below the tumour; upon loosening the tourniquet again, blood flowed from the tumour, but not so freely. The tumour was now detached still more from its bed; a knife-handle passed under its middle, and along it, one ligature conveyed above, and another below; these ligatures were directed in such a way as to insulate the tumour completely, by being tied above it and below it; the one below being drawn first was found to restrain the bleeding completely, but to make every thing secure, and to put the disease beyond any possibility of recurrence, the upper ligature was also fixed.

The vein which probably was the one that had been bled was seen in front of the sac adhering closely to it; it appeared to be almost obliterated below, and was very small above. There was nothing like a varicose state perceptible in it; so that if it had really been punctured, the wound had healed.

The sides of the tumour were very thick, and indurated, which will account for its not being entirely flattened or collapsed by pressure before the operation, and there was no coagulated blood. Whether it was formed by a dilatation of the artery, or by a cyst on its side, was not ascertained, from the obscurity of the parts during the operation.

---

* Ibid.

The tumour sloughed off kindly in this case, and the wound healed by the 20th of October, the recovery being perfect.

We cannot help thinking that an operation might have been dispensed with in this instance. We treated one quite like it by compression with success. A small circumscribed aneurysm of the brachial artery may in a great many cases be so cured. The operation is severe, and should be avoided if milder means will answer.

CASE II.—*Varicose Aneurysm of the Femoral Artery.—Operation followed by Mortification of the Limb and Death.*—Col. P., of Florida, ætat. 30, stature 6 feet 2 inches, usual weight 170 lbs., while engaged in the service against the Seminole Indians, was wounded, April 15th, 1837, by a ball accidentally discharged from a pistol which he wore under his waistcoat. The ball was rather larger than a buckshot (say 80 to the pound.) It entered the left thigh two inches below, and a little within, the anterior superior spinous process of the ilium, and ranging very nearly in a line with Poupart's ligament, came out on the inner side of the thigh a little below the scrotum.

There was profuse hæmorrhage, estimated at from 8 to 12 pints;—the patient fainted immediately and continued insensible till the next day. An extreme swelling of the thigh followed—numbness and irregular sensations, with some slight bleeding at intervals afterwards.

*July 8th,* 1837. The left thigh has a strongly pulsating tumor just below Poupart's ligament, and in the course of the femoral blood-vessels; the tumor appears to be two or more inches in its transverse diameter, length not so discernible; its pulsation is arrested by pressure on the femoral artery above; also, by pressing on the artery at a point corresponding with the track of the ball, and where there is a sensation imparted of a hole in the artery; the femoral vein feels enlarged as it goes under Poupart's ligament, and has a pulsation in it. The superficial veins of the leg are smaller than usual, the saphena scarcely perceptible. To the finger the tumour has the thrill and vibratory motion of varicose aneurysm, and a stethoscope applied to it conveys a noise like that of a distant waterfall or mill race, broken in upon by a pounding corresponding with the beat of the heart, and intermixed with a loud quick purring like that of a cat going to sleep. It is extremely sensible to pressure; the extremity is useless; the patient's common position is on his back, with the thigh in a state of semi-abduction, and brought a few degrees forward to loosen the parts at the groin.

The pulse of the wrist has a short globular stroke with a very distinct interval; a very strong pulsation is felt in the epigastric region extending to the umbilicus, a feeble wavy pulsation is also felt in the right femoral vein just at Poupart's ligament. The probality is, that the latter, as well as the epigastric pulsation comes from the arterial blood flowing from the left femoral artery into the vein, and thus giving a pulsation to the ascending cava and its branches. The patient was in a state of considerable emaciation, but in good spirits.

On the 11th, the femoral artery was tied immediately below the ligament of Poupart by Dr. T. Harris, assisted by Dr. Horner. The pulsation immediately ceased in the tumor, the epigastric pulsation also ceased, and that of the femoral vein of the other side; the pulse at the wrist made a longer and more sustained stroke.

The afternoon and evening were spent in high excitement and restlessness from violent pain flying to different parts of the limb and hip and finally settling in the incision, where the sensation was that of every thing tearing to pieces. The temperature of the limb was very low. The limb mortified below a line drawn obliquely six inches above the knee. On the 18th the mortification stretched beyond its previous boundaries, and advanced up the thigh. On the 20th, it was within a few inches of the groin. Then the scrotum was affected, and soon the gangrene reached the anterior superior spine of the ilium. On the

26th, the thigh was cut off through the mortified part, to diminish the fætor, &c. On the 30th the mortification seemed definitively arrested. It did not extend so high in the interior as on the surface of the limb.

On the 2nd of August, the ligature was cut away from the femoral artery, it being now the twenty-second day. "A whitish spherical tumor, an inch and a half in diameter, extremely sensible to the touch, but without pulsation, and which I had observed for a day or two, now attracted my attention particularly. I felt doubtful whether it was the aneurysmal tumor ; a cyst containing matter ; or a tumor formed on the course of the anterior crural nerve."

3rd. "Determined to explore the character of the tumor which had obtained our attention, I began to dissect from it the mass of mortified muscle at its inner side, and then made a puncture into it ; a flow of arterial blood immediately followed. I passed my finger into the orifice to check the hæmorrhage, and to obtain time for reflecting on its cause and the mode of proceeding. My first idea was, that I had acted prematurely in taking away yesterday the ligature from the femoral artery ; but from this scruple, I was relieved by ascertaining that pressure on the femoral artery did not stop the bleeding ; consequently, there must be a collateral supply of blood. I then determined to explore the whole circumference of the tumor, and to surround it in every direction with ligatures ; with that view, I cut through an isthmus of living matter, in front of the femoral vessels, consisting principally of fascia, and some adhering cellular substance. On doing which, it became clear that the tumor was aneurysmal, and that the disease had returned. I also perceived a very thin cul de sac, hemispherical, and half an inch in diameter, connected with the aneurysmal tumor and pulsating strongly. In the manipulations of the sac this tumor burst and bled freely. I then proceeded in tying in masses every thing connected with the sac, in consequence of my finding the structure so altered, and so many vascular connections of the sac, that the ordinary arterial arrangement was much modified, if not radically changed. I put ligatures above in the line of the femoral artery, another in the direction of the os femoris ; another in that of the pubes, and another between the most posterior face of the tumor, and the trochanter minor. The latter being done according to the suggestion of Dr. Goddard, who, during this time held the aneurysm in a state of compression which arrested the bleeding, and saved us from the distress of seeing our patient die on the spot.

These events were attended with a flow of blood from the original seat of the operation, and I then supposed that the artery had ruptured at the spot where its ligature had been, but when all the new ligatures had got their position, this bleeding ceased, so that the probability is, it came from a retrograde instead of a downward current of blood, and which was proved in the dissection after death. The obscurity of the vascular connections of the aneurysm, and of its internal arrangements compels me rather to conjecture than to state it as a point ascertained, but I am of opinion that the place punctured by me first was the enlarged femoral vein ; and the protuberance which ruptured was the aneurysmal sac. The principal part of the bleeding and the most rapid gush of blood was certainly from the latter ; the orifice of communication between the two vessels, was, therefore, still open as it had been."

By half-past 1 p.m. the patient had lost about a gill, or more, of blood. Dr. Horner opened the stump and tied some small arteries. The patient was in a deadly state. On the 4th, he had pain in the right side of the throat in swallowing. On the 7th, drenching perspirations commenced. On the 8th, the ends of three nerves are now exposed for an inch or two and are in a state of inflammation ; the sciatic ; the obturator ; and the anterior crural, all of which give excessive pain on the lightest touch. Now a bad ulcer began on the coccyx. Pain, too, was felt in right shoulder and in the hips. On the 9th, delirium ; small puddle of blood, of dubious origin, under his buttocks. On the 10th, the femoral veins seemed to be open. At night some hæmorrhage apparently from it. On the morning of the 12th he died.

The os femoris was in a state of necrosis up to the epiphysis formed by its head, and by the trochanter major. On being opened with a saw, a darkish serpentine line indicated the demarcation of the previously dead part; and the cells of the latter appeared to have undergone a limited suppuration. The bone being struck on its head, before it was sawed open, emitted a hollow sound. Some pus was found in the hip joint, the synovial membrane of which had a dark appearance. The anterior half of the cartilage of the os femoris was thinned by absorption apparently from the surface, which may have been done by the contiguous synovial membrane which covers the capsular ligament, as the cartilage from the fixed position of the limb had been, probably, since the original accident in contact with the synovial membrane; part of the cartilage in this region of the bone was entirely absorbed. The capsular ligament was sound.

The femoral artery just above the place of the original ligature was converted by its influence into a cul de sac; the bottom of which was firm, adherent, and about a line in thickness, and had a conical coagulum of bloody fibrine adhering firmly to it, about three lines long—with the apex upwards, and terminating at the orifice of a small artery, perhaps one of the external pudics. To this apex was appended a filament of the same fibrine, an inch or more long, running upwards in the canal of the artery.

Exactly where the ligature had been placed, the artery had been cut through by it, and the canal of the artery put on the appearance of a dilatation, and was continuous also with the original wound now reduced to the state of a small conical cavity. The artery was pervious from that to its inferior end. Trunk of artery thickened.

The femoral vein was open below; a probe passed unobstructedly from its inferior orifice upwards *sine limine* into the iliac. It was enclosed, as well as the iliac and the lower part of the ascending cava in an additional coat of tenacious fibrinous matter, which made them adhere firmly all along their course to the corresponding arteries and subjacent fascia iliaca. The external coat of the femoral and iliac vein was thickened and hard; and the internal coat exhibited the remains of strong inflammation, by its irregular slate-coloured surface, covered with a deposit of coagulating lymph. This appearance and deposit went up the ascending cava on its left side for two or three inches and there terminated in an angular manner. On the cava of this region were found some two or three plates, oval, half an inch in diameter, looking very much like the glands of Peyer in the intestines. Some lymphatic glands in a state of suppuration containing a small quantity of fetid slate-coloured pus existed in the course of the iliac vessels. No suppuration existed around the rectum and bladder.

From amongst several queries of Dr. Horner's, we quote the following.

1. "Did not the mortification ensue from its being easier, on the ligature being applied to the femoral artery, for the arterial blood to flow by the anastomoses of the obturator, gluteal or ischiatic arteries into the circumflex—thence into the sac, and return thence by the femoral vein, than flow to the foot and parts below the groin? The freedom of this anastomotic communication with the sac was proved, by the aneurysm continuing, though the main current of blood through the femoral artery was cut off, as proved by the necropsy. Would it not, therefore, be always proper to secure in every direction the arterial trunks, primary and collateral, communicating with a varicose aneurism, so as to force the blood downwards to the limb below, and thus prevent the possibility of its returning to the vein without a complete circulation?

2. Did not much of the extent of this mortification, at least in the thigh, depend upon the deleterious gas generated by it below, forcing itself upwards by emphysema under the skin, and between the interstices of the muscles? In those cases, therefore, is it not better to open freely the integuments and even the fascia to prevent such an accident, or to perform amputation ?"

9. " Ought we not, before obliterating a main arterial trunk, to spend, if it be at our option, some time in dilating the collateral branches, by pressure on the trunk itself, interruptedly applied ? This salutary practice appears to have fallen into unmerited disuse."

---

### Luxation of the Humerus during a Fit of Epilepsy. By W. A. F. Browne, M.D. Superintendent of the Crichton Institution for Lunatics. Dumfries.*

" G. R., aged thirty-one, a strong and robust man, and for many years subject to epilepsy, accompanied by mental derangement, upon one occasion, immediately after the cessation of a very violent fit, but before he appeared to know what he was doing, was observed to place his left hand upon his right shoulder, and heard to moan. He suffered so much from pain, and could so ill describe his sensations, that I was sent for. I found him crying piteously, grasping the shoulder, but otherwise unable to explain what was the matter with him. The testimony of all around (and several of the patients being convalescent, were credible witnesses) proved that he had not fallen—that he had received no blow—and that, in short, there was no cause known for his present state. I caused him to be stripped, and, upon examination, found that the humerus was luxated downwards and inwards. The bone was easily reduced."

Sudden violent contractions of the pectoralis major and latissimus dorsi are said to have luxated the humerus, when it was separated from the side. An instance has also been recorded in which the lower jaw was luxated during a fit of epilepsy, and remained unreduced ; and Dr. B. is informed that a case occurred many years ago in the Royal Infirmary of Edinburgh, in which, during an epileptic fit, *both humeri* were dislocated : the dislocations were reduced with great ease by Sir George Ballingall, who had charge of the patient.

---

### Scirrhus of the Stomach probably Congenital. By Thomas Williamson, M.D. Edin.

The subject of this case, a male infant, died at the age of five weeks. At birth, it was plump, and apparently healthy, but a few days afterwards vomiting came on, and the matter ejected was coagulated milk. During the last fortnight the bowels were obstinately constipated, and the child seemed to be falling off considerably in flesh, till it gradually sunk exhausted. Upon dissection, the intestines were found collapsed and empty, and all the other visceral organs perfectly healthy, with the exception of the stomach, the pyloric extremity of which felt hard and indurated, forming a remarkable contrast to the soft and yielding parietes of that viscus when emptied of its contents. Upon removing the stomach, it was found that the pyloric orifice was so contracted, as scarcely to ad-admit a small silver probe, a state which might perhaps in part account for its being nearly filled with coagulated milk. Upon slitting open the pyloric orifice, it was evident that the tissues entering into the composition of the parietes of the stomach, had greatly lost their normal appearance. The mucous coat was slightly thickened, whilst scarcely a distinct remnant of the middle or muscular tunic was observable. On the other hand, the sub-mucous cellular tissue

---

* Ed. Monthly Journ. of Med. Science, No. 1.

was so much hypertrophied and indurated, as seemingly to be the only tissue contained between the mucous and peritoneal coats : transverse white bands appeared to stretch from the sub-peritoneal to the sub-mucous cellular tissue, through that which formerly constituted the middle or muscular coat.

Both parents of the child were healthy, nor was there scirrhus in the family.

---

### ON BLEEDING IN MANIA. By W. A. F. BROWNE, M.D. Superintendent of the Crichton Lunatic Asylum, Dumfries.*

Dr. Browne points out the, in many respects, injurious circumstance, *that* the early acute stage of mania is generally treated by those who have no great practical acquaintance with the disease, and who likewise labour under the disadvantage of the patient being at home, without the *materiel* of a mad-house to assist them—while those who *are* acquainted with the disorder receive the patient only when it is confirmed. In nine cases out of ten, when a professional man, even of eminence, is called to a case of recent mania, he orders general depletion, the liberal exhibition of the solution of tartar emetic, brisk cathartics, and cold applications to a shaven scalp. If the symptoms, and especially the violence and rapid pulse, continue, or return in unabated force, the patient is perhaps bled again from the arm, or, if not, he will be cupped or leeched to a certainty. And after these energetic measures are manfully urged for days or weeks, and the tartar emetic lustration fails in every respect, except in producing a nausea-quiet, an asylum is recommended as the last resource. By such a step, the patient is removed from the observation of the practitioner, who can neither prosecute the principles upon which he founded his treatment, nor accurately watch the consequences of what he has already done; and thus he may naturally conclude, and often does conclude, that the subsequent treatment is but a necessary continuation or completion of what he commenced, and that, in fact, he has scientifically and successfully paved the way for regulating the more advanced stages of the malady. This is a gross and grievous mistake. When a patient enters an asylum, the plan adopted agrees neither in principle nor application with that previously pursued.

Bleeding, then, is *the* one remedy, which (in Scotland) seems to be applied to every madman. *Vide* these samples.

1. A strong, sturdy sailor, rendered insane by grief, restless, vehement, but abounding in generosity and good humour, alternately the queen of British tars, and the queen-mother of heaven, and a filter. Pulse 120, full, but soft, pervigilium; had been bled. 2. M.B. a slender, nervous female, incoherent, restless, sleepless, erotic. Pulse 90, strong; bowels constipated; tongue clean and moist; had been bled. 3. G. M. suffering from religious mania, combined with imbecility, bent upon the immolation of one of his sisters; reported to have had fits; had been bled. 4. D. C. seized first with stupor, then with acute mania, lastly with dementia. Pulse 80, soft, pupil of right eye contracted, insensible to light; had been bled. 5. A. B. a clergyman whose mind had given way under excessive exertion, he having preached seven times in succession; pale, thin, nervous; had been bled. We confess that we doubt the equal extent of this sanguinary practice in England, though it is pretty general too.

Depletion in derangement seems to have the following disadvantages :—1. It materially retards the recovery. 2. It gives a tendency to dementia. 3. It is sometimes directly fatal. 4. It debilitates at a period of depression, and in no

---

* Edinburgh Monthly Journal of Science, No. II.

degree facilitates the operation of other remedies. 5. It is dangerous during excessive muscular action.

1. Dr. Browne has found that cases in which general blood-letting has been employed, are more intractable, more obstinately resist the effects of other means, and are constantly of longer duration than those in which another course has been pursued. He presents a table of twelve recent cases, as similar as can be expected. Such have been selected as are cured, or curable, and as have not suffered in any other way from depletion. Of the twelve, six were bled, and six were not bled. Of the six not bled, all have recovered; of the six bled, three have recovered, and three are still under treatment.

The duration of treatment is as follows:

| Not bled. | | | Bled. | | |
|---|---|---|---|---|---|
| A. | 2½ | months. | B. | 4 | months. |
| D. | 6½ | —— | M. | 8 | —— |
| L. | 4 | —— | R. | 16 | —— |
| W. | 6 | —— | H. | 5½ | —— |
| W. | 2 | —— | N. | 14½ | —— |
| P. | 3½ | —— | D. | 20 | —— |
| | 24½ | | | 68 | |

2. Dr. Browne believes that dementia more generally follows where depletion has been pursued. He is the more inclined to think so from these facts:—that even in such patients as have been bled, but are ultimately cured, a stage of imbecility approaching to fatuity separates the period of excitement from that of convalescence. Dementia follows directly and obviously great evacuations and copious blood-letting, where no symptom of alienation pre-existed. There is a case under his care where incurable dementia succeeded the loss of blood in pneumonia. Hæmorrhage is likewise a cause of the same disease. There is under his charge a lady who is subject to periodical discharges of blood from the bowels, who is weak and imbecile during the attack, but is comparatively sane when it ceases. Individuals who have attempted suicide, by cutting the throat, and suffered from loss of blood, often fall into dementia.

3. He mentions several instances in which bleeding seemed to occasion fatal exhaustion.

4. In a vast majority of the cases admitted into asylums, it is necessary to order a generous and nutritious diet. In every case where bleeding has been resorted to, a full regimen is indispensable. Unless it be given, the patient is in danger of sinking from that vague, but well-known "exhaustion," which figures so largely in the bills of mortality of the insane, and which evidently depends upon the withdrawal of that nervous influence from the organs of assimilation, which an unimpaired cerebro-spinal and ganglionic system exercise, and which is essential to healthy nutrition. Nor until the loss of strength which succeeds bleeding, and which is of course aggravated by mental anxiety, be met, can active remedies of any kind be tried. Opiates act, it is true, with greater power; but even if their efficacy were greater than it is believed to be, their influence is very limited, when compared with that of the agents which are inadmissible from the want of strength. In a London asylum, where both bleeding and emetics were used upon a grand scale, it was found that when vomiting was induced after bleeding, apoplexy frequently followed; and it is instructive to learn, that of 200 bleedings drawn at the same time five only were buffed and cupped. The strong frequent pulse, which is supposed to indicate and justify this practice, is a most deceptive symptom. It is often not characteristic of the disease at all. When present, it is much more frequently the result of moral perturbation, than of a sthenic state of the brain, or any other organ. The constant agitation, the tremendous muscular efforts, in which the phrenzied strength develops itself, likewise contribute to keep up the pulse. The accuracy of this observation is shown by the rapid fall which takes place when the patient is compelled to take rest and repose. To bleed in such a case, in the expectation of reducing the momentum of the circulation, is vain and pernicious. To bleed, in order to subdue the violence of the mania, is more so.

5. Among the dangers may be enumerated the loss of large quantities of blood, after that prescribed has been withdrawn. This takes place from the difficulty of securing the vein, from the wound bursting forth during paroxysms of violence, and from the patient tearing off the bandages. We select two instances. 1. A medical man was largely bled during delirium, following a fall from his horse. During the night, he imagined that he was necessitated to walk out with two ladies, who appeared to stand at his bedside. In his attempt to dress, the vein began to bleed, and bled until he fainted. He passed from syncope to convulsion and died. 2. A carpenter was affected with violent suspicious mania. His friends had applied to a veterinary surgeon, who bled him profusely, and left him to the superintendence of his mother. Whenever reaction commenced he became doubly furious and unmanageable, tore the ribbon from his arm, escaped, was pursued, but continued to fly, although the blood flowed freely from his arm; and with such rapidity did he run, as to complete, three times in succession, the circuit of a carriage passing quickly upon the road. He was literally run down. He has now sunk into a state of imbecility and hebetude so profound, that all sensibility of the surface seems to be obliterated.

Dr. Browne has never seen a case in which local bleeding was not preferable to general.

---

### On the Employment of Opium for Arresting Acute Internal Inflammations. By Robert Christison, M.D., &c.*

I. The varieties of internal inflammation which best exemplify this action of opium, when given singly, are the inflammations of the mucous membranes. Of such diseases there are at least four which may often be thus successfully treated without almost any other remedy, namely,—coryza, catarrh, influenza, and dysentery.

"As to *Coryza,*—most persons, on feeling its approach, are content to submit without a struggle to the infliction, and eschewing alike physic and the physician, let their 'cold in the head' take its own way. But few would do so, were they aware how easily and how agreeably so tormenting a visitor may for the most part be got rid of. For twenty years I have been accustomed to see it stopped at once by a full opiate given on the first day of its appearance. Let the patient avoid food after dinner, use liquids sparingly, take a full dose of muriate of morphia, or Battley's solution, at bedtime, and breakfast before getting up next morning,—and he will then commonly find the secretion of the nostrils permanently inspissated, and the complaint either gone entirely, or at any rate no longer a source of particular annoyance."

This is no new practice to us. We have occasionally employed it on others, as well as on ourselves, for many years. We have usually exhibited the compound tincture of camphor in a large dose, half an ounce, or more. But we have taken care to give a purgative next morning, and, even with that safeguard, candour compels us to acknowledge that it has occasionally disagreed. It does *very* often stop coryza as by magic; but when it does not answer it may upset

---

* Edin. Monthly Journ. of Med. Science, No. II.

the stomach much. We cannot but consider Dr. Christison's picture as a little too gay.

Dr. C. has often seen a common *Catarrh without fever* cut short in like manner, if taken on the first, second, or perhaps even the third day. Or more generally it seems to pass at once over the intervening stages to that in which thick sputa are coughed or hawked up without labour, and without irritation in the chest or wind-pipe. *Febrile catarrh* too may be checked abruptly in the same way, if patient and physician are lucky enough to meet during the first or the second day at farthest. But here probably the next mode of employing opium is fully more successful.

In epidemic dysentery, Dr. C. believes that the cure may often be effected by opium alone, begun early and given boldly. In the epidemic of 1828, opium was given at once in the dose of one, two, or three grains every six, four, or three hours, according to the urgency of the symptoms, until signs of its narcotic action began to display themselves; and in this way sometimes twenty, or even thirty grains of opium were given in the first four and twenty hours. In several cases, where the treatment was begun not later than the close of the second day, the force of the disease was at once broken completely. And the only measures needed afterwards, were the continuance of the opium in diminished doses, and the restriction of the food to pulpy solids.

II. The method of using opium along with ipecacuan as a sedative, anodyne, and sudorific, in the early stage of acute inflammations, is probably applicable to a considerable number of diseases of the kind. Those in which Dr. C. has employed it are common sore-throat, catarrh, and acute rheumatism.

*Cynanche Tonsillaris.*—Dr. C. thinks that few cases may not be cut short if they are subjected to it about the close of the first, or, at all events, before that of the second day. Dr. C. relates two cases. We quote the second :—" A lady, about thirty-eight, long a martyr to cynanche tonsillaris,—which, in particular for three years before, had returned during the winter, ended in abscess of one or both tonsils, occasioned great and protracted torture, and had left chronic enlargement of these glands,—was attacked in the same way for the fourth time. When I first saw her on the fourth day of the disease, the tonsils, greatly enlarged, especially on the left side, blocked up nearly the whole throat. She spoke with much difficulty, could not swallow without long, painful, and repeated efforts, and breathed noisily and with labour. The pulse was 116, full and soft. The febrile oppression and anxiety were great. A large blister had been applied to the throat, and purgatives taken freely, but without the slightest benefit. The attack seemed too far advanced to be arrested. But as fluctuation could not be detected in the larger tonsil, Dover's powder was ordered, as in the former case. Sweating ensued ; ere long warm drink could be swallowed with no great difficulty ; and when the perspiration had been thus kept up for twenty-four hours, the pulse had fallen to 64, the swelling of both tonsils had materially subsided, and the febrile anxiety had ceased, together with the local uneasiness in a great measure. In two days more there was little complaint left except weakness. In the course of time, the chronic enlargement of the tonsils disappeared ; and this lady, like the last, has never had another attack, though five years have elapsed since the one now described. The tendency to cynanche tonsillaris certainly seems to grow with its repetition in a severe form ; and I have met with other instances besides those two, where an abrupt early cure seemed to break both the attack and the liability."

*Acute Rheumatism.*—" I am convinced," says Dr. C., " that, at least in young adults of sound constitution, a genuine acute attack may often be put an end to in a few days, by first drawing blood very freely to the approach of faintness, and then giving Dover's powder instantly afterwards in the way mentioned above for inflammatory sore throat. I have often observed, that after sweating has been thus brought out and kept steadily up for thirty-six or forty-eight hours, the pulse fell to the natural standard from nearly double that rate, the white thickly-furred tongue began to clean, the pains and redness gave place to numbness and want of power, recovery went on afterwards swiftly and without interruption, and the patient was able to leave his bed in a week. The chief conditions for success are, that the bowels, previously opened if necessary, shall be let alone till the sweating is well over; that blood be drawn both largely and to approaching syncope; that the powders be given immediately afterwards, before the circulation recovers its state of excitement, which it will otherwise soon do; that the treatment shall be enforced before the close of the fourth day, but earlier if possible; and that the case shall be real acute rheumatism, not one of the subacute form, or gouty rheumatism, as it is called,—where for some days previously the local inflammation has been shifting from joint to joint, with irregularly intermitting fever. I have once or twice tried to do without the preliminary blood-letting, but have not succeeded."

III. The treatment of acute internal inflammations by opium, after blood-letting, consists in withdrawing blood very freely from the arm till faintness approaches, just as in the ordinary way of treating acute inflammations,—and then giving a large opiate, with a view to bring on sleep or the calm reverie which in some people takes the place of sleep. The result is, that, on the patient awaking, the general fever and local inflammation are found to be subdued and broken,—generally at once, but sometimes not till the repetition of the practice in twelve or twenty-four hours. Dr. Christison cites several cases. We shall only pick out one or two.

*Case.*—" A hewing-mason, aged 45, a broken-down man for his time of life, and subject for some years to obstinate cough in the winter, was seized with symptoms of acute *Pneumonia* in the left side. There was crepitation in the lower part of that side, rusty sputa, hard cough, hurried breathing, and a frequent hard pulse. On the second or third day, when I was first called to see him, I took away towards eight-and-twenty ounces of blood, which brought on sickness; and he then got 45 minims of laudanum. The usual effects ensued: he awoke free in a great measure of pain, cough, and difficult breathing; but his recovery afterwards went on slowly. Next winter he was tormented to an unusual extent with cough, expectoration, and shortness of breath. In the subsequent winter these symptoms recurred again severely; and ere long acute symptoms supervened, which resembled acute bronchitis rather than pneumonia, and which, after being checked for a few days by the same treatment as on the previous occasion, acquired fresh force and proved fatal. This was obviously a case of acute inflammation, superadded twice upon the chronic affection known by the name of 'mason's asthma.'" We cannot say that this case is a very encouraging one. The next, perhaps, may be deemed more so.

*Case.*—" A lady, about thirty years of age, and subject to dyspepsia, being loth to lose the gay season of the year, neglected for three weeks a short tickling cough, with dull aching and obscure tightness in the lower part of the left side. At length she was seized with chilliness followed by fever, acute pain in the side, hurried breathing, and more frequent, short, dry cough. I saw her within twenty-four hours, and found the pulse 110 and sharp, the respirations about thirty-six, the left side circumscribed in its movements, the lowest third of it quite dull on percussion, and without respiratory sound, the middle third somewhat dull on percussion, with unequivocal ægophony, and the most remarkable crackling or friction-sound attending inspiration and expiration, which I ever heard. There was here much reason to dread chronic pleurisy, with a supervening acute form of the disease. In the evening a moderate blood-letting induced faintness; 35 minims of solution of muriate of morphia, given immediately afterwards, brought on quiet slumber; and next morning the fever and acute pain were gone, and the respiration was natural in frequency. The dulness on percussion in the lower part of the chest slowly decreased, and the crackling extended by degrees over the whole lower two-thirds of the side. This symptom continued for a week, during which every other complaint subsided; and she soon recovered completely."

Dr. Christison adds :—

"The conditions for successfully employing opium after blood-letting, are nearly the same with those for using Dover's powder in rheumatism. It is essential that the disease to be subdued be in its early stage; that a deep impression be made on it by blood being withdrawn both freely, and to the approach of faintness; and that the opium be given largely and immediately, so as to anticipate the renewal of re-action. Sweating sometimes ensues, but is not at all a necessary condition for success. The particular preparation of opium to be used is perhaps of no great consequence. I prefer the solution of muriate of morphia, or, failing that, the sedative solution of Battley. The dose of the former should not be less than forty minims for an adult male, and for others in proportion; and the dose of Battley's solution, which is certainly not so strong as its maker represents, should not be less than twenty-five or thirty mimims. Some conceive this treatment applicable only to inflammation of membranous surfaces, not to that of parenchymatous textures. I do not know any positive facts either on one side or the other of this question; but the statement is doubtful, if it be meant to apply to acute parenchymatous inflammations in their early stage. If pneumonia be regarded as inflammation of a parenchymatous tissue,—which, although a common mode of viewing it, is rather incorrect,—then, there can be no doubt that in this particular instance the treatment is most effectual on many occasions."

We confess that we are of the number who think opium should be employed in acute inflammations cautiously. A valuable medicine, beyond doubt, it is, but one sadly liable to be abused. And we fear that were the plan of treating acute inflammations with it very generally adopted, we should soon have a very respectable per contra sheet to oppose to Dr. Christison's flattering list.

---

### Immoveable Fracture-Apparatus for Varicose Ulcers of the Leg.

John Grant, aged 45, of spare habit, but of general good health, was admitted into the Leeds Infirmary, Sept. 8th, 1840, under the care of Mr. Teale, on account of ulcers of the leg.

After he had been purged freely and confined to bed for two days, lint was applied over the sores, and the entire leg from the toes to the knee was enveloped in the "immoveable fracture apparatus," consisting of calico stiffened with mucilage and chalk. The material employed for this purpose is similar to that which was stated to be in use at St. Bartholomew's Hospital, and consists of a kind of pasteboard, formed by two layers of thick calico cemented together by an interposed stratum of mucilage and chalk. Two portions of this pasteboard, adapted to the form of the leg, and softened by immersion in tepid water, are applied to the leg, and retained by a spiral calico bandage, over which is spread a layer of mucilage and chalk, and afterwards a second spiral bandage.

After the apparatus had been applied eight days, a slight degree of putrid odour was perceptible, and there was some appearance of matter oozing through the bandages at the situation of the ulcers; the apparatus was therefore removed, when the ulcers were found to be much diminished in size, the varicose veins no longer perceptible, and their thickened parietes could not be felt beneath the skin. The sores were dressed with lint, wax ointment, and a light bandage.

Oct. 9th. Ulcers healed. No return of varicosity.—*Provincial Med. and Surg. Journal. Nov.* 21, 1840.

---

### THE UVULATOME.

A novel and most ingenious instrument, invented by Mr. John Milliken, of Dublin, for excising any portion of the uvula which it may be desirable to remove. It is a forceps having a sliding blade on the upper surface. The portion to be removed is taken hold of by the forceps, and secured by a small bolt projected by the thumb; the fore-finger then projects the cutting blade, which cuts off the desired portion in a moment, which being held by the forceps is brought out of the mouth. This instrument renders the operation an affair of a few seconds—it is simple, ingenious, and effective. The inventor of the uvulatome has, we understand, several other new instruments in progress, which we shall have pleasure in introducing to the notice of the medical public on this side of the Channel.—*Medical Times, July* 4, 1840.

---

### DEATH FROM BRANDY AND SALT.

We are much disposed to agree with those who think it useless to legislate against quackery. When credulity can be put an end to by Act of Parliament, then, and not before, quackery can be stopped. But so long as there are rogues to promise impossibilities and fools to believe them, the charlatan's trade will subsist.

All our readers know that the dish of humbug that tickles the public taste just now is—brandy and salt. We dare say it will have its victims. Indeed here is one.

A man, advanced in years, had been the subject for many months of a tumor under the chin, for which he had received the best advice that could be procured in the neighbourhood, and was given to understand it was of a cancerous nature. He afterwards met with a pamphlet on Brandy and Salt, and soon began to try the remedy, taking it internally and *rubbing it on his head.* On the 5th of December he was attacked with hæmoptysis during a fit of coughing; and having lost a good deal of blood, he became alarmed and sent to me for assistance. When I arrived the bleeding had ceased, save an occasional streak of blood in the sputa, and his fright had in consequence passed away. I spoke to him seriously about his complaint, and was about to treat his case, when he told me he had been taking brandy and salt with great benefit to the tumor, and that he could not consent to leave it off; nor would he allow the application of any other remedial means, although I pointed out as strongly as I was able the danger he incurred by persisting in such a course. I was obliged to leave the house without doing any thing for him, but cautioned his wife (who also urged the continuance of the brandy and salt, thinking the loss of blood was some salutary discharge from the tumor, occasioned by the remedy) to watch him very closely, and not allow him to make use of any exertion. Soon after this, the patient paid his men's wages, went to bed, had a fit of coughing accompanied with another discharge of blood, and in a few minutes was a corpse.—*Prov. Med. and Surg. Journ. Jan.* 2, 1841.

---

Polypus of the Uterus removed by the Hand. By J. Toogood, Esq.

In June, 1830, I visited, with a gentleman of this place, a woman between 50 and 60 years of age, who had been suffering for a long time from violent hæmorrhage from the uterus, and on making a careful examination, a polypus of very extraordinary size was discovered. It was proposed to pass a ligature around it, but the patient wished to defer the operation for a short time, and when the attempt was made it was found impracticable, in consequence of the polypus being so soft and yielding, as to render it impossible to carry the ligature over its stem. As the patient's safety depended on the immediate removal of the tumor, I insinuated my hand into the posterior part of the vagina, in the hope of being able to place a ligature around it, until I found the stalk between my fingers; I then twisted it off, and withdrew the largest polypus that I ever saw; no hæmorrhage or bad symptom followed, and in a few days the patient was quite well.—*Prov. Med. and Surg. Jan.* 23, 1841.

---

Amaurosis and Night-Blindness produced by Onanism and inordinate Venery. By Robert Cane, M.R.C.S., Kilkenny.

Case 1. *Amaurosis from Venery.*—October 5th, 1839. J. D., aged 34, under the middle size, slight, but muscularly made, countenance flushed and drowsy looking; complains of sense of fulness in the head and dimness of sight, to such an extent that he occasionally walks with his hands stretched before him to avoid being knocked, and some weeks ago he walked into the River Suir off the quay of Waterford. The pupils are broadly dilated, the left most so; iris sluggish under light; conjunctiva and sclerotica are deeply injected; is slightly dyspeptic; bowels confined; pulse 90, and nothing unusual in its character; was in Stevens's Hospital in the early part of the summer, where he was put under mercury; had blistering ointment to his head, and a seton in his neck; thinks his sight was improved by the salivation, but it was as bad as ever in a few days after; but the mercury cured severe neuralgic pains in his arms and legs, which he then laboured under. The seton in his neck fell out, and he is now worse than when in Dublin.

This man's complaint is clearly traceable to his having married three years since, and as he states it, "having used sinful means to force, and enable him to gratify desires oftener than nature wanted." The practices were omitted, and the following treatment adopted :—

Cucurbitulæ C. nuchæ appl. et mittatur sanguis ad ℥xv.

℞. Pil. Coloc. c. gr. x. Ft. pil. ii. h. s. s.

The next day he felt much better; he continued under treatment until the latter end of November, in which time he was again cupped twice, had the seton replaced, and got aperients occasionally. He is now in the perfect possession of his sight.

There is another case, which we omit.

Case 2. *Night-blindness from Onanism.*—May 4th, 1840. J. Q———, aged 26, complains that he gets quite blind every evening as it grows dusk, and remains so until morning breaks; that he has been in ill health these twelve months past, but was not blind at night until about two months since; pupils natural size; iris slow under light; eyes and skin have a dull icteric tinge;

---

* Dublin Journal. November, 1840.

pulse 120, and feeble; feels weak, and has lost his appetite; tongue covered with a light brown coat, moist and flabby; had pain in the right side about three months back; right hypochondrium still swollen, and tender under pressure; abdomen tumid, but does not appear to contain fluid; the alvine discharges are scanty, but contain some bile; urine high-coloured and small in quantity. Has practised onanism for the last seven years, and to a frightful extent; has nocturnal emissions every night. Was directed to rub tart. ant. ointment over the right side, and to take night and morning the one-sixth of a grain of sulph. ferri, and 4 grs. of Rufus's pill, and to have porter and free animal diet. Under this treatment his general symptoms have improved; the enlargement in the right side is much lessened; the discoloration of the skin is disappearing, and he feels stronger, but the night-blindness still continues. Mr. Cane has commenced quinine with him, and he has given up his mal-practices.

Case 3. *Night-blindness from Onanism.*—May 20th, 1840. J—— K——, aged 18, complains he is blind at night; can barely see stars round a candle; feels weakly; but has no pain or sickness of any kind; is thin and emaciated; pupils a little dilated, and iris slothful; countenance pale; tongue moist, and, as in all those cases, morbidly soft and flabby; pulse 100, and feeble; bowels and kidneys acting healthily; admits that he has been practising onanism since he was fifteen years old; has often practised it four times daily—latterly not so often; nocturnal emissions; has felt the blindness creeping on him for the last year; sees perfectly well during the day. The treatment has been an aperient, quinine pills, and nourishing diet. He is rapidly improving; he can now see objects at night, but cannot yet see to read or work.

Mr. Cane presents us with the physiognomy of an onanist, or indulger in gross sensuality. There is, he says, a peculiar expression about the eye, partly caused by a corrugating of the lids at the outer canthus; a peculiar expression of the eye itself, a slow and stealthy mode of moving it over, and up and down an object, a language in the glance itself; while the lips are compressed and protruded, a "tout ensemble" not easy to analyze, but which the observer of human character soon becomes familiar with, and which, if his station or his profession be one permitting him to master men's secrets, will lead him to recognize a distinct order of the human family, many of whose diseases may be thus traced within a narrow compass, and to a positive source.

He believes that both diabetes and amaurosis are too often traceable to indulgences of this description.

"The first case of diabetes mellitus I ever witnessed occurred about the year 1826; that patient acknowledged to me that he dreaded onanism had caused his disease. Since then I have seen ten cases of diabetes; of that number six acknowledged onanism to its most destructive extent, one admitted it to the extent of using partially as a means to arouse passion for sexual intercourse, and the remaining three denied every thing of the kind, whether with truth I know not. But the admission of the other eight made a deep impression on my mind, and I cannot now meet with an amaurotic, or a diabetic patient, in whose case, if, after due investigation, some other cause is not discoverable, without my mind reverting to this, in my practice at least, most frequent source of disease."

We think Mr. Cane has done well to direct attention prominently to the consequences of these vices.

### On the Treatment of Various Diseases. By Robert J. Graves, M.D.*

### 1. *Looseness of the Teeth.*

Among the various causes which produce looseness of one or several teeth, none is more common than inflammation of the alveolar processes and sockets. Sometimes this originates in disease of the tooth itself, or of the gums; but in other instances, the diseased process commences in the alveolar periosteum, and by spreading to the socket and gums, it gives rise to great pain, swelling, and sponginess of the latter, while it eventually detaches the fangs of the teeth implicated in the attack, from the grasp of the sockets, and thus at last the teeth fall out, though in themselves they exhibit no appearance of decay.

The progress of the disease, is accompanied by extreme pain, and as a puriform discharge oozes out from between the gums and the inflamed periosteum, many limit their attempts to local means, and often succeed in effecting a cure by frequent applications of leeches to the inflamed gum, and in very obstinate cases, by incisions freely made through the gums and inflamed periosteum. Last year a patient of Dr. Graves' was thus affected, and thus treated, and although under the care of a most skilful surgeon, and of an eminent dentist, he lost successively a left bicuspis and molar of the upper jaw. His sufferings were for a short time relieved by the extraction of each tooth, but in a few days became as agonizing as ever, when finding all the neighbouring teeth loose, and being told that they also must soon be drawn, he had recourse, in despair, to a celebrated homoiopathic doctor, whose infinitessimal doses completely failed. Dr. Graves recollected that he had successfully treated him for a periostitic affection of the sternum and ribs, and that hydriodate of potash was the medicine which served him most. He recommended him to use ten grains of it three times a day, and had the satisfaction of perceiving a daily improvement, so that pain and inflammation soon ceased, and in about ten days the teeth were all fastened.

The periostitis to which this gentleman was liable, was of a rheumatic nature, otherwise his constitution was sound, and he was only thirty-four years old.

### 2. *Tinea Capitis.*

Dr. Graves makes some remarks on the dry scaly form. This species of ring-worm or dry tetter, is very contagious, and sometimes makes its appearance in one or several spots on the scalp, face, or other parts of the skin, but seldom is observed on the lower extremities or abdomen. It scarcely ever remains for any great length of time fixed in any part, except the hairy scalp, where it is apt to locate itself and become permanent. Dr. G. recommends attention to the following points.

"1st. When the disease is of long standing, always insert an issue in the arm before you attempt its cure. I have seen water on the brain, and other fatal consequences, from the neglect of this precaution.

2ndly. If this disease has clearly originated from contagion, and no other evidence of derangement of the general health can be detected, we must not, from the mere presence of the cutaneous affection, infer a constitutional taint, and must avoid the common error, of making the poor children undergo a course of alterative medicines.

3dly. This affection originating in contagious matter directly applied to the skin, cannot, like some varieties of lepra and psoriasis, (to which it often bears a great resemblance,) be cured by internal medicines, such as mercury, arsenic, and iodine, given separately or in combination, as in Mr. Donovan's new preparation.

---

* Dublin Journal of Medical Science, November, 1840.

4thly. When it occupies the hairy scalp, the common procedure of shaving the head is injudicious, for it adds to the irritation of the skin; and the scalp can be sufficiently exposed by cutting the hair as close as possible with a sharp scissors.

5thly. The great object is to get rid of the morbid action which is going on, and which consists in an inflammation of the external surface of the corium; an inflammation occurring in spots, and giving rise in the first place to an increased secretion of epidermis, which produces the scaly appearance of the parts affected; and in the second place, to a very slight and scarcely perceptible oozing of moisture which immediately dries into scales, and thus escapes notice, being mingled with the scurf formed by the detached portions of morbid epidermis.

6thly. The cure must be accomplished by removing these scales, as far as that can be done by diligent ablution, without using any irritating degree of friction; and when the diseased portion of the skin has been thus exposed, we must next have recourse to some application which will destroy the morbid secreting surface. Formerly this was attempted by means of an endless variety of complicated formulæ, each of which had its advocates; the list may, however, be now reduced to a few simple remedies, and in truth, with nitrate of silver, sulphate of copper, or strong tincture of iodine, every case of this disease may be cured.

7thly. I never use the solid lunar caustic, or sulphate, but prefer a solution of ten, fifteen, or twenty grains to the ounce as the case may require. As to the application of this solution, it will not do to apply it, as is generally done, with a camel's hair pencil, *for it must be strongly rubbed into each spot,* for which purpose a small bit of sponge, covered with fine linen, and tied to the end of a quill or slender stick, should be employed. When a large portion of the scalp is affected, it will require some perseverance to apply this lotion in an effectual manner.

8thly. An application of this nature, when effectually done, must not be repeated oftener than once a week.

9thly. Immediately after it the whole scalp must be covered with a spermaceti dressing, and the spermaceti must be renewed at least four times daily, so as to keep the head constantly moistened with it. The head is not to be washed for three days after the application of the caustic, or of the tincture of iodine, but then it may be well but very gently washed with yellow soap and water twice a day, taking care to cover, as before, with a spermaceti dressing after each washing.

In scaly diseases of the skin, it is quite surprising how much the cure is facilitated by keeping the affected parts constantly smeared with spermaceti, oil, melted suet, or even candle grease. Without this aid, the use of caustics will often disappoint the practitioner.

10thly. When the above precautions have been taken, the cure will advance rapidly, and each succeeding application of the caustic solution, or of the tincture, may be less severe."

### 3. *Hydriodate of Potash in Sciatica and Lumbago.*

"I first became acquainted with the remarkable efficacy of this medicine in lumbago and sciatica, under the following circumstances. In the memorably wet month of July, 1839, I was called out of bed at midnight, to visit a lady in the country, and the vehicle sent to convey me was a hack covered car. The cushions were very damp, and I had not proceeded half a mile, before I was attacked with lumbago, so severe, that I could scarcely walk when I arrived at my patient's residence. Next morning I was better, having perspired much during the night; but still the pain was troublesome, and as the season continued unusually cold and wet, (indeed it scarcely ever stopped raining from the 8th of July, 1839, to the 19th of February, 1840,) and as my duties exposed me much to the weather, and prevented me from giving myself the necessary rest, my lumbago continued to increase again, and in about a month, the gluteal and sciatic nerves of the left side became engaged; I noted particularly, that the pain spread very gradually downwards from the lumbar region, so that it took a week or ten days to arrive at the ham, and a still longer time at the ankle; I was then quite lame of the left leg, suffered much from pain in bed, and had become so helpless, that I had to get my servant to draw on my stockings; during all this time my general health was perfect; appetite good; digestion regular; and no deviation of the urine from the natural appearance. I mention this, because several of my medical friends advised me to take antibilious aperients, an advice founded on Abernethy's doctrine, that many local affections proceed from stomach derangement. I was at last forced to try something for my relief, and had myself cupped, and tried the warm douche and Dover's powder, but without any good effects. I began now to fear, that I should be forced to give up all professional business, and confine myself to the house for many weeks in order to go through a mercurial course, combined with proper topical applications, when happening to meet Mr. Ferguson of Kildare-street, he recommended me to try hydriodate of potash, of which he was good enough to send me a drachm dissolved in a pint of decoction of sarsaparilla. I took quarter of this daily, and may literally apply here the common phrase, that I felt each dose do me good; in truth the benefit I derived was perceptible hourly, and was so rapid, that in four days all traces of the lumbago were gone, and my lameness had quite ceased. I did not take more than one bottle, i. e. one drachm of the hydriodate, but the good effect continued after I had ceased taking it, and in less than a week, I was perfectly well. Subsequent experience enables me to recommend this medicine strongly, in subacute and chronic lumbago and sciatica.

It is right to observe, that the remedy had in my own person to work against various disadvantages, for I neither relaxed from my labours, nor refrained from eating and drinking as usual. This is only another example of the many I have met, which prove how injudicious it often is, to seek the cure of local inflammations by means of lowering the whole system."

### Inoculation after Vaccination.

Dr. Blennerhassett, of Tralee, has communicated to the Dublin Journal a paper on Small-pox after Vaccination, from which we are tempted to extract some rather curious particulars.

About the autumn of 1835, the small-pox was introduced into Dingle and the neighbouring villages, by one or two itinerant quacks, who inoculated the children of the poor at a shilling a-head. Dr. Blennerhassett's children had had the cow-pox perfectly. But in November two of his sons were attacked with small-pox, and one had it in a confluent and severe form. He now determined to inoculate the remaining nine, with matter taken from the first, who had the small-pox mildly.

"In eight of these the progress of the small-pox inoculation was as follows:—On the second or third day, a small vesicle appeared with the edges red, and somewhat inflamed from the first, presenting pretty nearly the appearance of the spurious cowpock; it was not so circular as the vaccine pustule, and had not the flat top, which I reckon the chief distinctive mark of the latter. The pustule increased in size, and the angry, festered appearance of the edges became more apparent, till the eleventh or twelfth day, when the erysipelatous inflammation, though not quite so vivid and well defined as in cowpock, encircled the pustules. This inflammation, in some of them, went quite round the arm, occupying about half of it; and in one boy of eleven, it fairly surrounded the entire arm from the top of the shoulder to the elbow, in the shape of a slight *erythema*, so that I thought it right to apply a saturnine lotion, which soon subdued it. This inflammation disappeared in all of them in two or three days, leaving a larger sore than in cowpock, which soon became incrusted with a black scab, which continued several days; and in one or two, when it was rubbed off prematurely, exhibited a deep red pit, and on healing, left a deeper eschar than in cowpock. About the fourth or fifth day a slight swelling in the glands of the axilla of the affected arm was perceptible, with considerable pain on pressure, or on moving the arm.

On the sixth or seventh day all the eight suffered a smart febrile attack with some rigors, increased heat, accelerated pulse, pain in the head, limbs, and back, and sickness of the stomach, with prostration of strength, and great depression of spirits; this continued two or three days; but in a lad of seventeen and the boy of eleven, the fever continued for five days so extremely severe, as to confine them to their beds, and I began to repent that I had not let them take their chance, apprehending that this fever would be succeeded by a crowded eruption; however, I had the pleasure to find, that the febrile symptoms gave way without the eruption of a single pustule, excepting a number of minute vesicles which surrounded the inoculated part of the arm. The child in whom the inoculation failed was an infant whom I vaccinated three months before; I repeated the inoculation, however, in the summer of 1836, and he then took it in the form I have now detailed."

He was now pressed to inoculate ten members of a family, which he did with matter taken from his son who had had confluent small-pox. He found the result almost exactly similar; the symptoms of pain in the axilla, and the occurrence of fever on the sixth, seventh, or eighth day, proving that the constitution was fully affected; one only of this family resisted the inoculation, a girl of about fifteen.

"Besides the nineteen now recounted, I inoculated fifty-one more in the course of a few weeks, the majority of them being children of the gentry of the town and neighbourhood, of various ages, from that of five or six to puberty; the fever and other symptoms (except the pain in the axilla, which was sometimes wanting) were the same as already detailed; in the case of one young lady, the fever appeared so early as the fifth day; but on whatever day it appeared, it was generally sufficiently active to confine the patients to the bed, for two days at least, when it gradually subsided, as in my own family, without any decided pustular eruption, except in two instances, in each of which, a gentleman and lady of about twenty, somewhat more than 200 perfect small-pox pustules occurred in the face and body. When variolous inoculation was practised previous to the introduction of cowpock, the progress of the punctured vesicle, as well as the pain in the axilla and febrile symptoms, were very nearly similar to those I have now described; and in a great majority of cases, particularly in those who had undergone a preliminary preparation for inoculation, there occurred no pustular eruption, except the minute pustules which surrounded the areola of the punctured part. The small-pox, therefore, in sixty-eight out of seventy of my cases, was, I almost venture to say, as fully communicated, as if the patients had never been vaccinated."

---

### Introduction of Syphilis into the System, through other Channels than Sexual Intercourse. By Clement Hamerton, Surgeon to the Castletown Dispensary.*

Mr. Hamerton relates some cases, from which he draws the following conclusions:—

---

* Dub. Journ. March, 1841.

A healthy child is applied to the breast of a venereal nurse, in a couple of weeks syphilis shews itself in the child. A venereal child is applied to the breast of a healthy woman, soon afterwards she gets a syphilitic sore of the breast, which contaminates her system. A servant girl sucks a venereal sore breast, she gets a venereal ulcer of the mouth, which taints her system. The midwife has a slight scratch on the palm of the hand, and in delivering a putrid venereal child, she gets a sore on the hand which infects her system; and lastly, the husband of the midwife is diseased at the time the ulcer exists upon his wife's hand. The husbands of Duffy and Fay had not a single syphilitic symptom, nor did they take any mercury. The only inference I think warranted from the above cases (differing from the received opinions) is, that when the death or disease of the children has been caused by a syphilitic taint in the mother, not received from the male parent, it is not necessary to subject the latter to a mercurial course.

---

### Cracked Tongue.*

A country lad, aged 18, consulted Dr. Godson, on account of a deep groove, or crack, extending from the root to the apex; the edges of which groove or crack were somewhat elevated, rough, and irregular. The lad declared that he had suffered greatly from this affection during the last six or eight months; and that he had consulted several medical men, but had derived no benefit from their advice.

"Of course, I looked to the stomach (the unfortunate organ to whose impertinence so many local diseases are speciously assigned) as the proximate cause of the lad's 'bad tongue.' Accordingly, I set about putting the digestive organs into 'good order,' and applied various caustics to the part affected, &c.; but at the end of two months the groove or crack in the tongue remained in *statu quo*."

At the end of two months, the lad was no better. Dr. G. quotes the case of Charles Matthews, related in his "Memoirs." "In a few weeks (says Mrs. M.) after these harassing struggles, my husband found an occasional inconvenience, that he had lately felt, augmented to a most serious disorder, his eventual sufferings from which were truly pitiable. I can only describe it by saying, that it showed itself in *deep cracks across his tongue*. Every advice was sought and attended to; but it baffled the first-rate skill and experience. It sometimes prevented him from eating, and banished sleep; and had he not been resolute in the prosecution of his duty, he must have declared it (as his medical men did) impossible to use it professionally. Every word he uttered was like a drop of aquafortis upon these cracks. This complaint had in turn been pronounced to be *stomach* and *local fever*, caused by anxiety and his great professional exertions. On the days of performance he often found it requisite to preserve a total silence until he began his 'entertainment,' when he described his sensations to be like what he must be supposed to feel while talking and singing with a piece of red hot iron attached to his tongue."

This is not a very uncommon affection. It is a very troublesome one. As a general rule, solutions of caustic as applications, and sarsaparilla with the iodide of potassium or the nitric acid, as internal remedies, combined with regulation of the secretions, and the avoidance of mercury in anything like full doses have, in our hands, answered best.

---

* Lancet, Sept. 1840.

### DR. GREGORY *versus* "VARIOLÆ VACCINÆ."

We are come or coming to a happy state with reference to the protective power of vaccination. Our readers recollect that it has been just settled that cow-pox *is* small-pox. Dr. Gregory is quite positive that it is *not*. We quote from that capital little work—Braithwaite's Retrospect—a work very much wanted in these days of Journalism.

Dr. Gregory writes to Mr. Stuart :—

Will revaccination protect, and for how long? The true answer I believe to be as follows :—The value of revaccination is in one sense proportioned to the effect produced. If revaccination produces a full eight-day pock with areola, it stands *loco primæ vaccinæ,* and the individual may be said to open a new policy of vaccine insurance, dated from that period. On the other hand, if the revaccination produces little or no effect (a mere irritated papula), nothing is taken by the motion. The individual remains in *statu quo ante* revaccination. But then comes the question ; will a modified effect serve to fill up the measure of vaccine protection decayed during the preceding ten, fifteen, or twenty years ? This is the pinching part of the question. My persuasion is, that you cannot thus multiply degrees of vaccine protection. Two imperfect vaccinations do not, in medical arithmetic, equal one perfect one ; no,—nor three, nor four, nor twenty. Modified or imperfect revaccinations, therefore, in my estimation, = 0 ; they are worth nothing. They irritate the arm, and that is all. The constitution is uninfluenced by them. I may be wrong in this, and I am ready to correct the error, if it can be shewn to be error ; but all my experience goes to this. The doctrine of proto and deuto-vaccinations will soon merge in that of trito, and ultimately (as time creeps on) in poly-vaccination. Will a man be perfectly safe (that is to say) who is vaccinated (or subject to vaccination) every year? Those who support the present fashionable theory and practice of revaccination will please answer this question.

I have now brought you to the point which I have been anxious to gain. I have never yet addressed any one, in writing, on this subject, and I now write to you on it, because I see that you have considered it well; that you have thrown off the trammels of Jennerian pathology, and are content to think for yourself. Observe, I say Jennerian pathology, not Jennerian practice. I feel assured you do not view vaccination as a kind of small-pox. The term *variolæ vaccinæ* was incorrect in pathology. Cow-pock is a something that alters the human blood, and indisposes it to take small-pox. But it is not small-pox. A coating of gold secures our salt spoons from the action of chlorine ; but gold is not chlorine. Small-pox after vaccination is not on a par with double small-pox. Small-pox after vaccination is a first attack of small-pox, and may be followed by a second some twenty or thirty years hence. Well, then what is to be done to fortify the public mind in the matter of vaccine security ? How long are we to go on thus showing annually or epidemically our practical distrust of vaccination ? The sooner we come to a decision on the subject the better. There is one, and only one way in which this can be done. Not by revaccination, but by inoculation at distant periods from the date of vaccination.

Now as to the real pathology of vaccination. Jenner's theory must be given up, viz. that cow-pox is only small-pox in its mildest and most original form ; that a man has cow-pox now instead of the small-pox which he had formerly ; that small-pox occurring after cow-pox is analogous to small-pox after small-pox. These three positions, the foundations of Jenner's notions, are, I believe, entirely groundless and imaginary. The true pathology of vaccination is altogether different.

Now, vaccination has the extraordinary power of giving to the human body the singular property of resistance to the variolous effluvium. Vaccination is not small-pox, but just the reverse,—the antagonist principle.

What wonder, therefore, can it be, if time should demonstrate, that the power of resistance thus conferred, is confined within certain limits; as thus:

1. The power of resistance is complete (both as to casual and inoculative admission) for the first ten years of life.
2. The power of resistance ceases in certain constitutions before it ceases in others.
3. The power of resistance given by cow-pock ceases *quoad* inoculation before it ceases *quoad* the casual (or infective) mode of access.
5. The power of resistance is diminished, by any great changes taking place in the human frame, whether brought about by puberty, or change of climate, or by a long fever; or lastly, by gradual and insensible changes taking place in the system.

There may be many more laws affecting the general principle of resistance given by vaccination hitherto unsuspected. I believe there are. I see daily facts inexplicable on any known principle. For I wholly throw out of consideration Jenner's fanciful notions of vaccination impeded by pre-occupation of the skin; of vaccination rendered imperfect by the virus being taken at too late a period: or the more modern notion, that too few vesicles are raised; or those few not developing a sufficient amount of constitutional fever. All this is mere unauthorized theory. Two or three good vesicles on the arm constitute vaccination. When small-pox subsequently occurs, do not then reason *ex post facto,* and say the vaccination was faulty, but find out what are the laws which limit the principle of vaccine resistance.

Jenner set us all so wrong by his term *variolæ vaccinæ,* that it is really difficult to get out of the false (because so well beaten) track. If he had wanted a short expressive term, it should have been *vaccinia antivariolosa.* We should then have set ourselves to study how far the antivariolous power extended, and by what laws it is limited.—*Braithwaite's Retrospect.*

---

### Explanation of the Symptoms of Emphysema of the Lungs, by the Nature of the Anatomical Lesion.*

We extract the following from a paper on Emphysema of the Lungs, by Dr. Goolden. The paper gives an account of the views of Dr. Lombard, of Geneva.

The symptoms may be divided into two groups.

The one is the immediate consequence of the retention of air within the lung. The other is produced by the obstruction of the circulation, and inaptness of the diseased portion to perform the function of respiration.

To the first group belong—

1. The sonorousness of the chest; since a considerable quantity of air remains confined within the lung.
2. Distortion of the chest. It is a common observation, that the walls of cavities mould themselves to their contents; and if the lung continues permanently in a state of distension, the chest will be developed in the same proportion.
3. Absence of the respiratory murmur, the natural consequence of the fulness of the lung, which, being already distended to the utmost, can receive no more at each inspiration.
4. Atrophy of the inspiratory muscles, which Dr. Stokes has remarked as the consequence of the forced extension in which they are maintained by the morbid distension of the lung, and which may in a measure account for the weakness of the inspiratory sound.

---

* Med. Gazette, Jan. 29, 1841.

To the second group belong—

1. Palpitations.
2. Hypertrophy and dilatation of the right ventricle.
3. Dropsy, resulting from the obstruction to the pulmonary circulation through the morbid lung.
4. Dyspnœa, where there is a greater supply of venous blood than the diseased lungs can arterialize. This explains the various causes producing a paroxysm of asthma; such as exercise, mental emotion, stimulants, which increase the circulation, a full stomach, the recumbent posture, preventing the descent of the diaphragm, obstructions of the air-cells or lobes, which prevent the lungs from using their full power in performing the respiratory function.
5. The frequency of pulmonary catarrhs, the result of the increased activity of the healthy parts of the lung, in those patients in whom a whole lobe, and frequently a whole lung, is perfectly useless for the purpose of respiration.

---

### A MODE OF INTRODUCING THE CATHETER IN DIFFICULT CASES.*

Dr. Patterson has adopted a plan of introducing the catheter in cases of difficulty, which he thinks may be useful. We confess that we believe practice and dexterity are *the* things needful. But to Dr. Patterson's method. It consists in attaching a bladder of water to the catheter, and, when there is an obstruction, directing a stream through the instrument upon it.

He attaches a small bladder to the end of the catheter, after the manner of a mounted enema pipe, the bladder need not be large, one that holds about half a pint of water will be sufficiently so; and the catheter must be furnished with a small cork, having a piece of twine fixed to it, just like the cork of an enema pipe. The largest sized catheter the urethra will admit of should be used, that the impetus of as large a jet of water as possible may act on the obstructed part of the canal, and also that the urethra being filled by the instrument, the fluid may be easily prevented from returning by the side of the catheter. The eye must be large, and very near the end of the tube; or, what is better, the tube should have a large orifice within the circumference of its anterier extremity, with the edge rounded in so as that it shall not hurt, or catch the lining membrane of the passage. Also it must be a silver or permanently curved elastic catheter: for the injecting apparatus would prevent the withdrawal of the stilet necessary to maintain the curvature of a common elastic one.

The instrument, having the attached bladder properly adjusted, is to be passed down to the obstruction: if it cannot now, on further trial, be made to enter the patient's bladder, it must be held by an assistant, while the small cork is being inserted into its outer end: about six ounces of lukewarm water are to be poured into the injecting apparatus; the latter must then be closed, and tied as near as possible to the fluid, so as to exclude every portion of air. The operator is next to encircle the penis with the finger and thumb of his left hand, making gentle circular pressure to close the urethra round the catheter; he will then, having first withdrawn the cork, embrace the bladder of water with his right hand, so as to be able to apply strong and uniform pressure on as much of its surface as possible, in doing which, its contents being continuously and forcibly propelled into the urethra, and the handle of the instrument being at the same time depressed, the latter at once passes with facility into the patient's bladder.

The left hand in holding the penis, should be quite passive, allowing the ut-

---

* Dublin Journal, March 1841.

most freedom of motion to the catheter; the right hand should not touch any part of that instrument, but manœuvre it through the medium of the attached bladder of water, in the act of compressing the latter, so that its extremity may be free to follow, as it were spontaneously, the course the current of water opens before it.

### Miscellanies.

### Medical Reform.

